# High-Throughput Label-Free Continuous Quantification of Muscle Stem Cell Proliferation and Myogenic Differentiation

**DOI:** 10.1007/s12015-025-10915-7

**Published:** 2025-07-03

**Authors:** Stig Skrivergaard, Martin Krøyer Rasmussen, Margrethe Therkildsen, Jette Feveile Young

**Affiliations:** Department of Food Science, Aarhus University, Aarhus, Denmark

**Keywords:** High-throughput screening, Image-based cytometry, Label-free imaging, Satellite cells, Myogenesis, Myotube quantification, Live cell assays

## Abstract

**Background:**

Quantifying muscle satellite cell proliferation and differentiation is crucial for applications in muscle regeneration, disease modeling, and cultivated meat research. Traditional fluorescence-based assays, while sensitive, are labor-intensive, endpoint-restricted, and disruptive to myotube integrity.

**Methods:**

In this study, we present a novel high-contrast brightfield (HCBF) imaging technique for high-throughput, label-free assessment of both satellite cell proliferation and myogenic differentiation. Using the BioTek Cytation 5 automated imager and Gen5 software (Agilent Technologies), we optimized imaging parameters to achieve continuous, highly time-resolved quantification in standard 96- and 384-well formats without any additional reagents or cell manipulation needed.

**Results:**

Our approach enabled detailed kinetic profiling of satellite cell behavior, revealing myotube formation dynamics, species-specific media responses, optimal seeding conditions and the influence of mechanical factors on differentiation. We also demonstrated that serum-free media formulations could support efficient myotube formation in both bovine and porcine satellite cells, while having very different myotube kinetics and morphology than serum-containing samples. Furthermore, we highlighted the high degree of well-to-well variation and the sporadic formation and detachment of myotubes in culture, and the interesting phenomena of a second wave of myotubes being formed following detachment in serum-containing samples. Additionally, the 384-well format enabled a label-free screening method to assess clonal myogenicity of isolated satellite cells.

**Conclusion:**

By eliminating the need for genetic labeling, invasive staining or specialized consumables, our high-throughput HCBF methodology advances myogenic research, offering new opportunities for efficient screening and highly detailed kinetic data acquisition for serum-free media development, drug discovery and pathophysiological testing for both cultivated meat and musculoskeletal research.

**Graphical Abstract:**

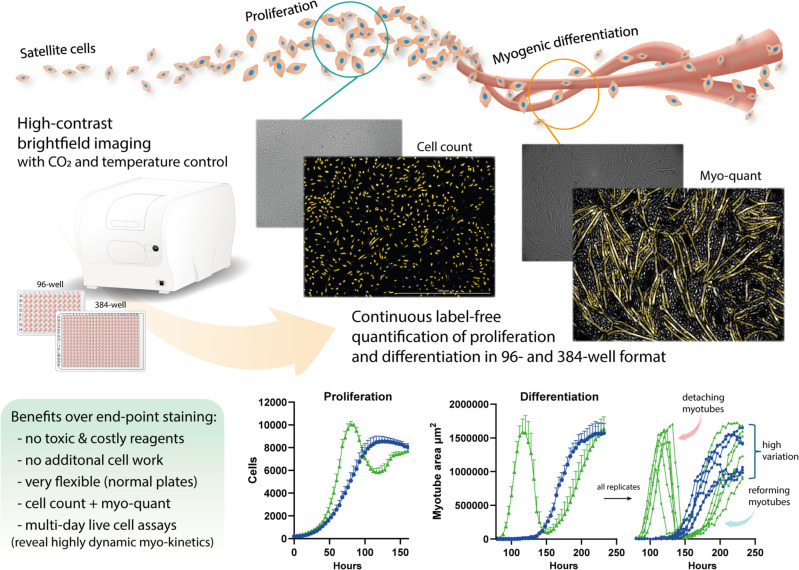

**Supplementary Information:**

The online version contains supplementary material available at 10.1007/s12015-025-10915-7.

## Introduction

Muscle stem cell culture optimization and knowledge generation is an essential part of both cultivated meat production and musculoskeletal research. In cultivated meat, muscle satellite cells (SCs) serve as the foundation for muscle fiber formation, offering a sustainable alternative to conventional meat with reductions in land use, water use and global warming potential [1]. However, key technical challenges still remains [2, 3]. In musculoskeletal research, in vitro cell models enable the study of muscle regeneration, disease mechanisms, and drug screening related to conditions like muscular dystrophy and sarcopenia [4]. SCs undergo a dramatic morphological transformation during myogenic differentiation, transitioning from proliferating myoblasts to mature muscle fibers under specific conditions [5]. Understanding this intricate process is crucial for studying muscle tissue regeneration and muscular dystrophies [6, 7], as well as developing cultivated meat from muscle cell cultures [8, 9]. Surprisingly, very few methodologies exist to efficiently investigate the dynamic growth and differentiation behavior of satellite cells [10].

High-throughput image-based cytometry and automated image analysis is a powerful combination in terms of gaining insights from cell culture, enabling efficient drug discovery, pathological screening and media optimization [11, 12]. At its core, image-based cytometry is all about extracting complicated phenotypic information and reducing them to simple quantifiable terms, which can be performed under normal cell culture conditions with obvious benefits over flow cytometry [13]. With the rapid technological evolution in automated microscopy and their expanded commercial availability, it is now possible to acquire intricate high-content cell data in a high-throughput format. The cellular parameters investigated can vary in complexity from the number of cells, their size and morphology, to intracellular protein expression and localization, cellular kinetics, and behaviors, which analysis has greatly benefitted from machine learning, software algorithms and AI [14–16]. Our lab has previously demonstrated the powerful analytical capabilities of the BioTek Cytation 5 multi-modal automated microscope (Agilent Technologies) regarding both satellite cell proliferation and their myogenic differentiation [12, 17].

The common practice of end-point fluorescence imaging for myogenic differentiation quantification typically involves laborious and costly procedures such as antibody staining for myosin-heavy chain and desmin [9, 18, 19], or the slightly easier staining of the F-actin with fluorescently probed Phalloidin [17]. In terms of proliferation, the precision and ease-of-use of Hoechst-stained nuclei counting cannot be understated, especially when compared to metabolic assays. However, the main caveat of these staining methods is their end-point nature due to the use of cytotoxic compounds and cell fixation needed. This makes it less suitable for dynamic proliferation assays and kinetic output, which would require many parallel cell seedings dependent on the desired temporal resolution. This is a very significant limitation especially when investigating events like very sporadic formation of myotubes which also tends to easily detach in culture or during the staining procedures [7]. Few non-destructive methodologies exist that enable repeated measures over time with kinetic data acquisition such as impedance-based real-time label-free differentiation monitoring [20] and myotube contractability measurements [21, 22], however these are very intricate setups that require specialized cell culture plates and devices which are difficult to implement in high-throughput formats.

Several label-free imaging techniques exist, such as brightfield imaging, Zernike’s qualitative phase contrast microscopy (ZPCM), differential interference contrast (DIC)/Nomarski microscopy, electrical cell impedance sensing (ECIS) and quantitative phase imaging (QPI) methods, yet only brightfield imaging is considered to be widely applicable with standard culture vessels [23]. Quantification of cells using brightfield imaging normally relies on the principle of defocusing microscopy [24] in which the cells themselves refract the light with a lens-like effect creating easily quantifiable bright spots [25]. This principle was first exploited in a high-throughput manner more than 10 years ago [26], and is now a common feature amongst several commercial automatic imager systems on the market. One such system, the BioTek Cytation 5, specifically make use of a high-contrast brightfield (HCBF) imaging technique that significantly increases cell contrast over normal brightfield imaging using a pinhole aperture combined with a defocusing strategy [27, 28]. With the use of the non-invasive label-free HCBF imaging technique the same cell samples can be quantified as many times as needed for highly time-resolved growth data with less labor, material use, and technical variations [27]. The Cytation 5 can specifically utilize HCBF imaging together with the CO2- and temperature-controlled environment, which enables long-term live assays with incredible temporal resolution in the kinetic growth data that is acquired. However, until recently cell proliferation was the only useful output from this imaging technique, yet now we present an extended application of HCBF which enables the label-free quantification of myogenic differentiation over time.

We have previously presented this novel label-free approach to quantify myotube formation [17], albeit only in a limited manner using 48-well plate format and two time-points. This was achieved by increasing the defocusing further than usual, i.e., imaging with a higher negative z-axis value relative to the in-focus image, to enhance the contrast of specifically the larger myotubes rather than single cells. In this research, we present an improved and more thoroughly tested methodology which enables proper high-throughput quantification of both proliferation (cell count) and myogenic differentiation (myo-quant) in 96-well and 384-well formats in a continuous label-free manner using HCBF imaging. This unobstructive, efficient and automatized imaging and subsequent image analysis by the Cytation 5 imager and Gen5 software (Agilent Technologies) enabled highly time-resolved data collection of the very intricate cellular dynamics of myogenic satellite cells which we utilized to optimize several serum-free culture conditions for both bovine and porcine SCs as a proof-of-concept.

## Methods

### Materials and Reagents

The serum-free media (SFM) used in this study was previously described [12] and consisted of 2 ng/mL FGF2, 600 µg/mL fetuin, 75 µg/mL BSA and 1 × ITS supplement in DMEM/F12 basal media with 1% antibiotics (penicillin 100 units/mL, streptomycin 0.1 mg/mL, amphotericin B 2.5 mg/mL). Specific reagents used are ITS (Gibco, 41400045), DMEM (61965–026, Gibco), DMEM/F12 (Gibco, 11330032), FGF2 (Future Fields, Bovine FGF2, EntoEngine, purified version), BSA solution (Sigma, A8412), fetuin powder (Sigma, F2379). Fetuin should be sterile filtered directly after solubilization in DMEM/F12 using a 0.2 µm PES membrane (Sarstedt, Filtropur S, 83.1826.001). DMEM/F12 was also used in all 10% FBS (fetal bovine serum) control samples. Proliferative growth medium (PGM) consisted of DMEM (GlutaMAX, Gibco, 10566016) with 10% FBS, 10% HS (horse serum), 1 mM sodium pyruvate and 1% antibiotics (penicillin 100 units/mL, streptomycin 0.1 mg/mL, amphotericin B 2.5 mg/mL) with gentamicin added (0.1 mg/mL). All chemicals used for cell culture work such as DPBS, trypsin and media components were from Gibco™ (Thermo Scientific, Rockford, IL, USA), except gentamicin and penicillin/streptomycin antibiotics which were from Sigma (Saint Louis, MO, USA). All other chemicals used were from Sigma unless otherwise stated. Standard cell culture plasticware was from Nunc (Thermo) with the Nunclon delta surface.

### Cell Culture

Bovine satellite cells (BSCs) and porcine satellite cells (PSCs) were isolated from the *M. semimembranosus* from a month-old Holstein bull calf and a Duroc Danish Landrace Yorkshire crossbred (approx. 20 kg), respectively, obtained from Aarhus University Viborg, Denmark. The muscle was dissected and transported on ice within 2 h to the cell lab at Department of Food Science at Aarhus University. The isolation of bovine and porcine SCs from the muscle tissue was previously described [29, 30]. The SCs were initially cultured in Matrigel coated T25 flasks (Nunc, 156367) for 3 days before being expanded in Matrigel coated T75 flasks for 2 passages (Nunc, 156499) using PGM before cryopreservation in PGM with 10% DMSO in liquid nitrogen. Cryopreserved cell samples (passage 2) were quickly thawed and diluted in PGM, before being pelleted at 500 × g for 10 min at 4 °C to remove DMSO-containing freeze media and resuspended in 37 °C PGM. SCs were then cultured in Matrigel coated T75 flasks for 2 days using PGM at 37 °C in a 5% CO_2_ humidified atmosphere before cell experiments. Matrigel (Corning, 354234) was diluted 1:50 in DMEM (defined as 1x) before 40 µL/well (96-well format) or 20 µL/well (384-well format) was added and incubated for one hour, before seeding cells for proliferation and differentiation assays. Vitronectin (VTN-N, Gibco, A14700) 1 × concentration was defined as a 5 µg/mL concentration.

### Hoechst-Based Nuclei Counting

BSCs were washed twice with PBS without calcium and magnesium (Gibco, 14190144) and detached with 0.25% Trypsin (Gibco, 15090046) in PBS before being collected in DMEM 10% FBS and centrifuged at 500 × g for 10 min at 4 °C. Cells were resuspended in DMEM/F12 with 10% FBS and counted using a Countess3 (Thermo). SCs were cultured in Matrigel coated 96-well (Nunc, 167008) or 384-well plates (Thermo, 164688) at varying cell densities and were allowed to attach in DMEM with 10% FBS for 24 h. The media was discarded before adding 50 µL/well of a 20 µM Hoechst 33342 solution (Thermo, 62249) (diluted in PBS) and incubated for 5 min. Hoechst solution was then discarded, and 100 µL/well PBS was added before plates were imaged using the BioTek Cytation 5 (Agilent Technologies) with DAPI laser/filter cube settings (4 × objective). The cellular analyses using Gen5 software (Agilent Technologies) were performed using a stitched montage of the entire 96-well (cropped to exclude the auto-fluorescent edges). Analysis parameters: signal threshold = 4000, split touching object = on, object size = 8–40 µm, rolling ball (RB) = 20 µm.

### HCBF Cell Count and Myotube Quantification in 96-Well Format

BSCs or PSCs were detached and counted as described above and then seeded in 100 µL/well DMEM/F12 with 10% FBS in 96-well Matrigel coated plates at a density of 1000 cells/well. PSCs were additionally seeded at 5000 cells/mL. After 24 h of cell attachment wells were washed with PBS and 150 µL/well of the experimental media types were added. As Cytation 5 does not have a humidified environment, the evaporation of liquid is imminent over time at 37 °C. Therefore, we recommend not using the edge wells but instead filling them with liquid (200 µL/well) and using at least 150 µL of media per well for assays longer than 3 days. HCBF imaging was performed in 4-h read intervals for several days depending on the live assay setup using the CO2-regulated Cytation 5. Using the “discontinuous” option in the kinetic settings enabled pausing and re-starting the experiment, allowing for other critical cell assays to be performed in between read intervals. Note that this is not an intended feature but currently works in Gen5 version 3.14 and 3.15. Instead of doing a focused image and then de-focus as previously [12], the de-focused image is made directly by adjusting the z-axis offset. This way the time and file size are effectively halved. The de-focused image is still plenty useful for qualitative assessment of cell morphology and behavior. The defocus z-axis settings for HCBF imaging were 2400 µm for cell count and 2070 µm for myotube quantification (myo-quant). Both HCBF cell count and myo-quant analyses were based on 5 × 4 images per 96-well using the 4 × objective (using overlap for montage/stitching). The 10 × objective can be used to fully image the entire well (12 × 10 images per well) without the meniscus effect, albeit at the cost of 6 × longer imaging time and 6 × larger file sizes. The Gen5 pre-processing parameters used was (both cell count and myo-quant): RB = 20–40 µm, quality = fine, image smoothing = 10. Analysis settings for the cell count was; signal threshold = 3000, split touching objects = on, size = 10–80 µm, RB = 20 µm. Analysis settings for the myo-quant was; signal threshold = 800–1200, split touching objects = off, size = 110–10000 µm, RB = 20 µm, evaluate background on % of lowest pixels = 60. Plugin area was circular at 4700 × 4700 µm with per experiment offset adjustments to center the plugin which is affected by plate displacement. The myo-quant defocus and analysis parameters that we used were based on extensive experimentation and can be used as a starting point for other researchers. These settings also work with much higher defocus setting (z-axis = 1670 µm), which can alternatively be used for more robustness yet with less myotube detail and inclusion (example shown in Sup. Figure 2 A). We recommend always thoroughly investigating the robustness of the analysis on a per-experiment basis and if needed optimize settings for the specific cell culture in question.

### HCBF Cell Count and Myo-quant in 384-Well Format

BSCs were detached and counted as described above and then seeded in 70 µL/well DMEM/F12 with 10% FBS in 384-well Matrigel coated plates at a density of 600 cells/well (or otherwise stated). After 24 h of cell attachment wells were washed with PBS and 80 µL/well of the experimental media types were added. Due to evaporation, we did not use the two outer rows/columns of wells but instead filled them with liquid (100 µL/well). All four tested 384-well plates had some type of quality issue/visual artefact within the plastic. The specific issues with each can be seen in Sup. Figure 7. The two Thermo plates tested, transparent (Cat. No. 164688) and black (Cat. No. 142761), were not suitable for HCBF myo-quant. The VWR (Cat. No. 10814–226) or Greiner (Cat. No. 781091) plates also had minor issues, yet both should be suitable for both cell count and myo-quant, although the VWR plate issue might require data analysis adjustment for the cell count only. HCBF imaging was performed at 4-h intervals with the discontinuous option as described above. HCBF z-axis defocus settings were z = 2285 µm for cell counting and z = 1580 µm for myo-quant. Pre-processing and analysis parameters were the same as described for the 96-well HCBF imaging. For the one-cell-per-well experiment the HCBF cell count and myo-quant were both based on the same HCBF image using the myo-quant defocus z-axis setting. The seeding density of 0.85 cells/well used for this experiment was chosen to reduce the probability of wells with more than one cell and would as per the Poisson distribution roughly correspond to ~ 43% empty wells, ~ 36% wells with 1 cell, ~ 15% wells with 2 cells, ~ 4% wells with 3 cells and below 1% of wells having 4 or more cells.

### Immunostaining

BSCs were seeded at a density of 500–2000 cells/well in a Matrigel coated 96-well plate using 10% FBS DMEM/F12. After 24 h of cell attachment, the samples were washed with PBS and 150 µL/well of either SFM or PGM was added. Cells were allowed to differentiate in PGM over a 4-day period and in SFM over a 7-day period. After myotube formation, the cells were fixed by 4% paraformaldehyde (PFA) for 10 min and permeabilized by 0.1% Triton-X for 15 min. After washing cells with PBS they were incubated in a 1% (w/v) BSA DPBS-Tween 20 (0.1% v/v) blocking solution for 1 h at room temperature. Phalloidin-AF647 (1:400) (Thermo, A30107) or primary anti-myosin heavy chain (MHC) antibody (M4276, Sigma) diluted 1:400 in DPBS-Tween 20 (0.1% v/v) with 0.1% BSA was added and incubated overnight at 4 °C. The MHC-stained cells were washed three times with DPBS-Tween 20 before secondary anti-mouse Alexa Fluor 488 (A28175, Thermo) 1:1000 was incubated for 2 h at room temperature. The last 15 min of incubation Hoechst 33342 (Thermo, 62249) was added in a 1:1000 concentration in PBS. Cells were washed three times with DPBS-Tween 20 and PBS were finally added before Cytation 5 imaging using the 4 × objective with DAPI, GFP and Texas Red filter/laser settings, accordingly. Imaging of the entire well area was automatically stitched before the myotube area was automatically obtained through an optimized Gen5 cell analysis.

### Statistical Analysis

GraphPad Prism 9 software (La Jolla, CA, USA) was utilized for graph generation and statistical analysis. One-way or two-way ANOVA with multiple comparisons (Dunnett’s or Tukey’s, respectively) were used to compare media types or groups when applicable with *p* < 0.05. All graphical data was presented as the mean value ± SD, except the HCBF live assay data in which we utilized ± SEM error bars for clearer graphic visuals.

## Results

### Label-free Cell Counting in High-throughput Formats Using HCBF Imaging

**Fig. 1:**
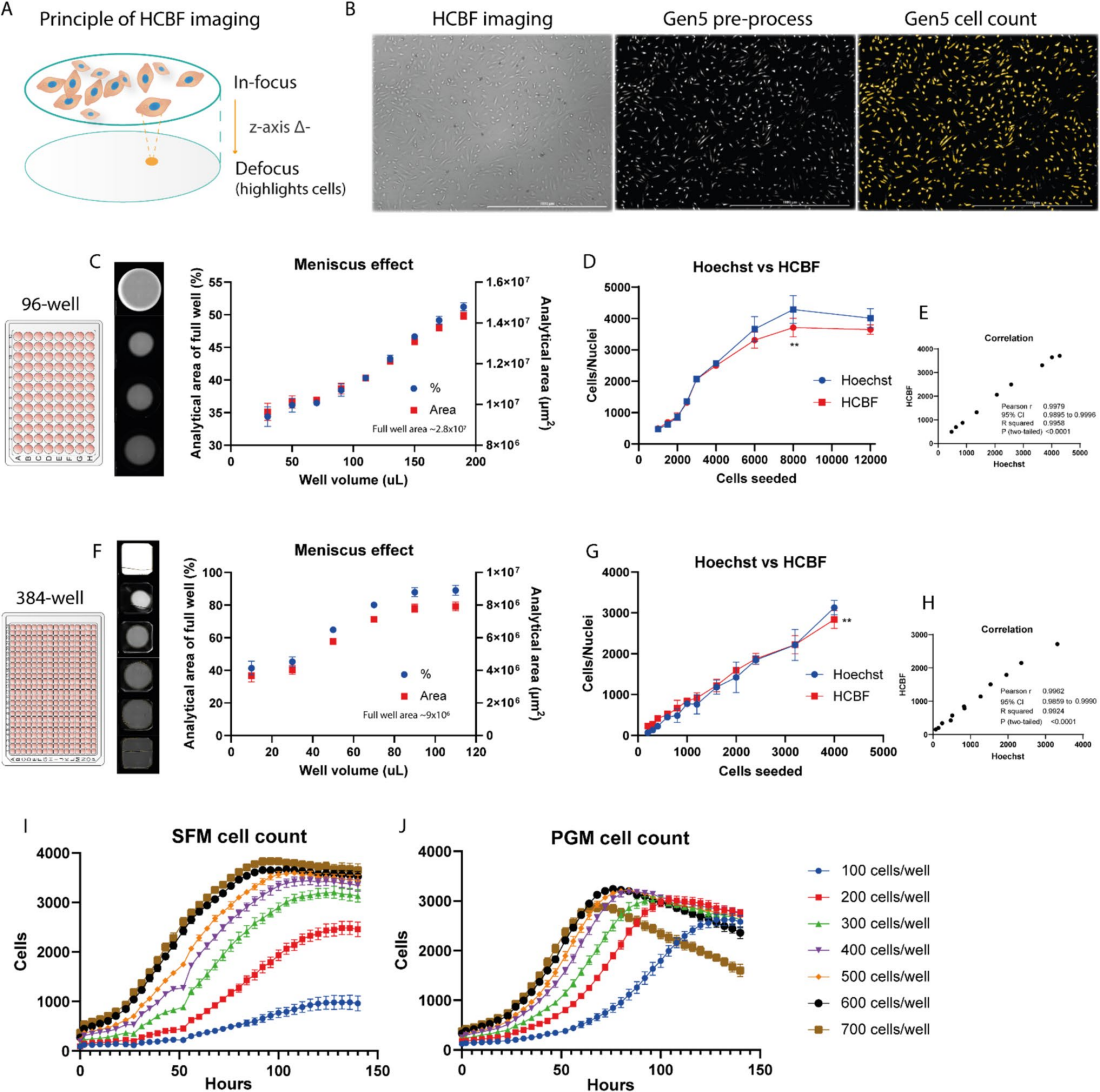
High-contrast brightfield (HCBF) imaging for the quantification of BSC proliferation in high-throughput formats. **A** Graphical representation of the Cytation 5 HCBF imaging technique in which the negative z-axis plane in relation to the in-focus image generate the defocused image with bright cell spots thereby increasing contrast **B** for Gen5 pre-processing and cell analysis. **C** Meniscus effect in the 96-well format varies depending on well volume and determines the analytical well area of HCBF imaging (*n* = 4). **D** Comparison between Hoechst-stained nuclei counting and HCBF cell counting at different cell seeding densities in 96-well format (*n* = 4) with the corresponding Pearson correlation **E**. **F** Meniscus effect in the 384-well format (*n* = 8). **G** Comparison between Hoechst-stained nuclei counting and HCBF cell counting at different cell seeding densities in 384-well format (*n* = 6) with the corresponding Pearson correlation **H**. **I + J** Live assay with continuous HCBF cell counting over several days in 384-well format with different seeding densities in either serum-free SFM (I) or serum-containing PGM (J) media (*n* = 6). Data is presented as mean ± SD (or SEM in live assay graphs). Statistical significance by two-way ANOVA with multiple comparisons in which *p* < 0.01 (**)

We previously demonstrated the reliability and usefulness of the continuous label-free high-contrast brightfield (HCBF) imaging to obtain highly time-resolved cell proliferation data [12, 17] in which the imaging principle behind this technique is illustrated in Fig. 1A and B. However, as that was only performed in a 48-well format, we aimed to validate the technique in more high-throughput formats, namely in 96-well and 384-well formats. Due to the much smaller well surface area in these formats we investigated how much the expected liquid meniscus effect would negatively impact the actual analytical area in HCBF imaging (Fig. 1C + D). In the 96-well format the meniscus effect was only allowing below 50% of the well area to be analyzed in the normal liquid volume range (100–150 µL), while the 384-well format had above 60% analytical area when liquid volume was 50 µL and increased significantly beyond that. Due to the observed meniscus effect and the liquid evaporation during longer assays we used 150 µL for the 96-well (~ 47% = 1.3 × 10^7^ µm^2^ analytical area) and 70 µL for the 384-well (~ 80% = 7.13 × 10^6^ µm^2^ analytical area). The meniscus effect could however be nearly negated using the 10 × objective (Sup. Figure 1 C) although with the large caveat of ~ 6 times longer imaging time and ~ 6 times larger file sizes due to the needed 120 stitched images to cover the full well area versus the 20 images needed with the 4 × objective for a 96-well. As long-term live assays in these high-throughput formats already have massive file sizes (> 200 GB) when using the normal 4 × objective we chose not to use the 10 × objective for more efficient data handling, while the 4 × objective still gave adequate analytical area when using the liquid volumes of 150 µL and above.

To assess the precision of HCBF cell counting of bovine satellite cells (BSCs) in the high-throughput formats we compared it with the very sensitive Hoechst-based nuclei counting at differing seeding densities (Fig. 1E + F). There was no significant difference between the two techniques in a broad range of cell numbers up until confluence for both formats in which HCBF cell counting would only underestimate cell numbers slightly. This data also showed a strong linear correlation, with Pearson coefficients of > 0.99 (with R^2^ > 0.99) for both the 96-well and 384-well plates (Fig. 1G + H), indicating high agreement between the two methods. This high correlation was also observed when using the 10 × objective (Sup. Figure 1 A + B).

To validate the HCBF cell counting in a more relevant setting we performed a live assay measuring the proliferation of BSCs at differing starting cell densities over a 140-h period in the 384-well plate format using either serum-free media (SFM) (Fig. 1I) or 20% serum-containing media (PGM) (Fig. 1J). The highly time-resolved data acquired showed very different cell growth kinetics depending on the starting cell density. The HCBF imaging can also be quantified in a manner of cell coverage area instead of cell numbers (Sup. Figure 1D) which shows the same growth patterns. At the lower cell seeding densities (100 and 200 cells/well) the growth seemed to be delayed with reduced growth rate and much lower maximum cell density at the plateau, especially noticeable in the SFM samples (Fig. 1I). Contrary to this, there was no visible difference in the growth kinetics when increasing cell numbers from 600 to 700 cells per well in both media types, in which the SFM achieved higher maximum cell numbers at the plateau, while PGM achieved its maximum earlier with significant decrease in cell numbers thereafter especially at 700 cells/well (Fig. 1J). This lower maximum cell density in PGM is due to a higher degree of cell spreading, i.e. larger surface area of cells, and because of the morphological change throughout myogenic differentiation which happens earlier in the serum-containing media. This was also reported on in our previous work [12]. The myogenic differentiation is also the reason behind the decreasing cell numbers as cells are undergoing fusion and myotube formation, which will be further highlighted in the next section.

### Label-free Myotube Quantification in 96-well Format Using HCBF Imaging

**Fig. 2:**
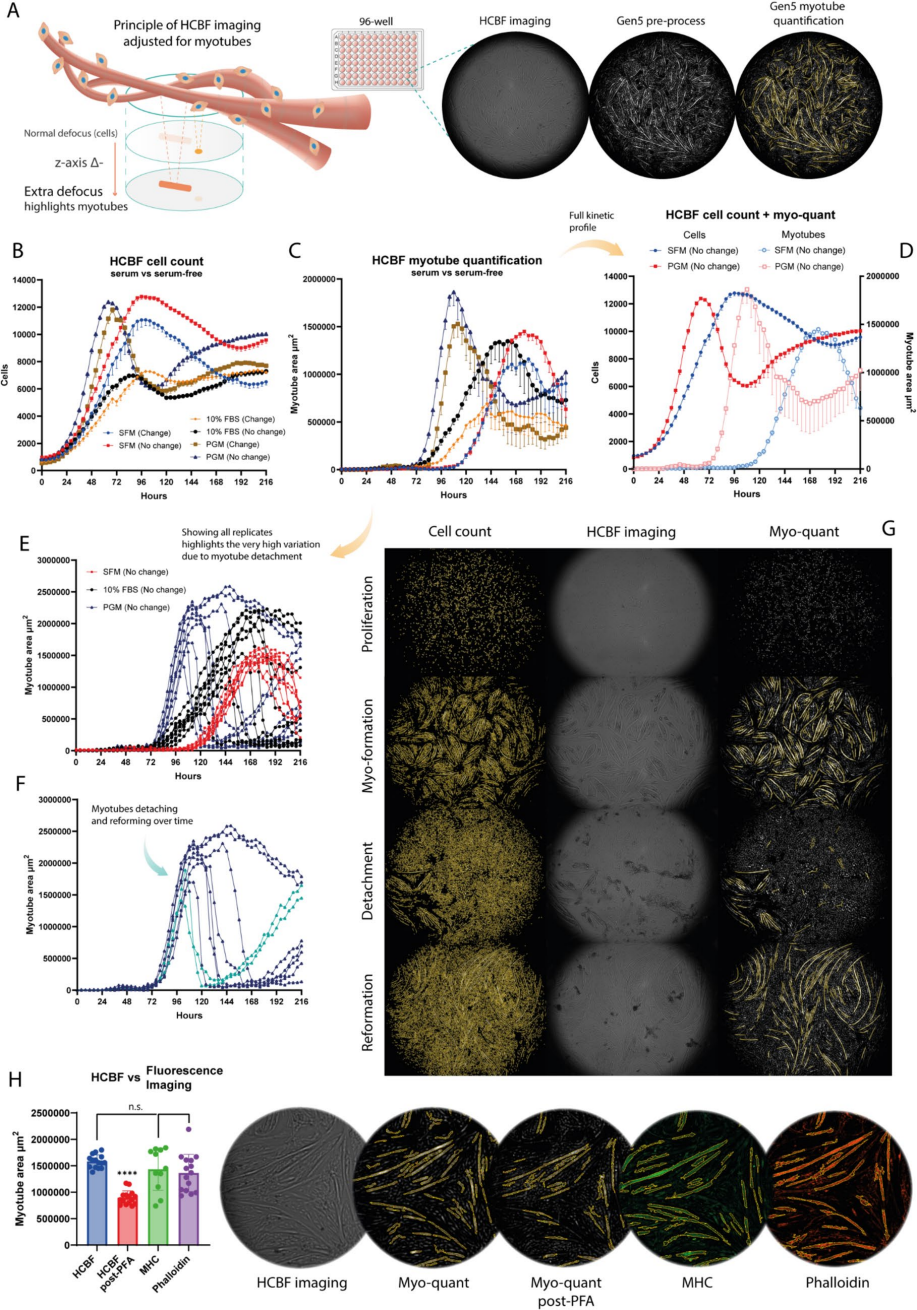
High-contrast brightfield (HCBF) imaging for myotube quantification in 96-well format. **A** Graphical representation of the HCBF imaging in which the negative z-axis plane is further reduced (relative to cell counting setting seen in Fig. 1A) to generate an extra defocused image which highlights the larger myotubes contra singular cells thereby increasing contrast for Gen5 pre-processing and myotube quantification in a 96-well format. **B** Live assay HCBF cell count of BSCs with either SFM, 10% FBS or PGM media, with or without a media change after 72 h (*n* = 6–9). **C** Same live assay as (B) with HCBF myotube quantification (area in µm^2^) of BSCs undergoing myogenic differentiation. **D** Combined graph of HCBF cell count and myotube quantification (myo-quant) for the SFM and PGM media types, without media change, for better visualization of the full growth and myo-kinetic profiles. **E** Showing all the individual well replicates from (**C**) highlights high degree of variability due to myotube detachment. **F** Only replicates from the PGM (No change) group are shown highlighting the two replicates with early myotube detachment and very high reformation of myotubes. **H** Comparison of HCBF myo-quant, MHC (myosin-heavy chain) and Phalloidin stained fluorescence imaging for the quantification of myotube area in SFM samples (no media change) after 7 days of incubation (*n* = 15), with representative images of the Gen5 analyses. Data is presented as mean ± SD (or SEM in live assay graphs). Statistical significance by two-way ANOVA with multiple comparisons in which *p* < 0.0001 (****)

We previously introduced the novel technique of adapting HCBF imaging to quantify myogenic differentiation, albeit only in a limited extent using a 48-well plate format and without long-term continuous imaging[17]. The principle in increasing the HCBF contrast of the myotubes rather than single cells is to increase the defocus, i.e. decreasing the z-axis value even further from the defocused plane of the normal HCBF imaging, as well as having optimized Gen5 pre-processing and analysis parameters (Fig. 2A).

Therefore, to test the full potential of this technique we designed an experiment to fully quantify the complete proliferation and differentiation of BSCs in one single live assay in a 96-well format (Fig. 2B-D). Two different HCBF imaging channels (defocused and extra defocused) was applied in the assay to continuously count the cells (Fig. 2B) and quantify the myotube formation (Fig. 2C) over a 9-day period when using either SFM, 10% FBS or PGM as growth media. Initial myotube quantification assays (Sup. Figure 2B + C) had indicated similar myotube formation without any media change when using SFM, and the three media types were therefore also tested in two different settings, either with a media change after 72 h or without any media change throughout the entire experiment. For the serum-containing samples the media change was a serum-reduced (5% FBS) media while for the SFM samples it was a reduced SFM consisting of only DMEM/F12 and ITS supplement based on initial results (Sup. Figure 2B). With the simultaneous cell count and myo-quant we observed very different kinetic profiles depending on the media type, with the fastest cell growth and differentiation obtained with PGM (Fig. 2B + C). When media was not changed the SFM resulted in similarly high cell densities as the PGM, at the plateau, though approximately one day later, while the differentiation was quite significantly delayed in SFM. The 10% FBS samples had by far the lowest rate of proliferation, yet initiated differentiation much earlier than the SFM samples which was improved with not changing the media. With all three media types it was evident that simply leaving the growth media throughout the entire assay had positive effects, especially in terms of differentiation, which can simplify experimentation and reduce media costs. When combining the cell count and myo-quant in one graph (Fig. 2D) it appears that the drop in cell numbers from the plateau signifies the start of differentiation as it almost perfectly matches the timing of the myotube increase. Furthermore, the well-known phenomena of myotube detachment can also clearly be seen with a significant drop in the myo-quant after a short plateau, which again is reflected in the cell numbers in this case increasing due to the newly formed empty surface area that can be re-grown after myotube detachment. Interestingly, when looking at the myo-quant data for PGM there seems to be an increase at the end of the assay after the myotube detachment and generally a very high replicate variation beginning after the myo-quant maximum.

We therefore dissected the data by showing all the individual replicates to better understand these myo-kinetics (Fig. 2E + F). In the comparison between SFM, 10% FBS and PGM (Fig. 2E) it is very evident that the serum-containing samples vary greatly in their replicates in terms of myotube detachment timing, whereas the SFM replicates seem to be more consistent in their detachment timing. In the 10% FBS and PGM samples, there are even a few replicates without any noticeable detachment contributing to the high degree of variation seen in Fig. 2C. A comparison of media change and no change samples of PGM and SFM with all replicates can be seen in Sup. Figure 2D + E. Surprisingly, the two earliest detaching PGM replicates (highlighted in Fig. 2F) also reforms their myotubes to a similar extent as the first round, again increasing variation between replicates and adds to the overall complexity of long-term differentiation kinetics. The simultaneous cell count and myo-quant for one of these replicates can be seen in Fig. 2G (or as a full live assay video in Sup. Vid. 1), in which the reformed myotubes can be seen to have an altered morphology.

To test whether the HCBF myo-quant numbers reflected the well-established method of immunofluorescence-acquired myotube quantification, we compared HCBF imaging against myosin-heavy chain (MHC) and Phalloidin staining. In SFM samples, myotube quantification was very comparable with no statistical difference between the three methods (Fig. 2H). The HCBF imaging contrast was, however, significantly affected by PFA-fixation and permeabilization as part of the staining protocol, which indicates the necessity to perform HCBF imaging on fresh/live samples. As a result of the more complex spread-out morphology of PGM myotubes, the HCBF myo-quant seem to generally underestimate myotube area when compared to MHC (Sup. Figure 3 A + B) and Phalloidin (Sup. Figure 3 C + D) stained samples. The very different myotube morphology of PGM and SFM samples can be seen in Sup. Figure 3E.

### Label-free Myotube Quantification in 384-well Format Using HCBF Imaging

**Fig. 3:**
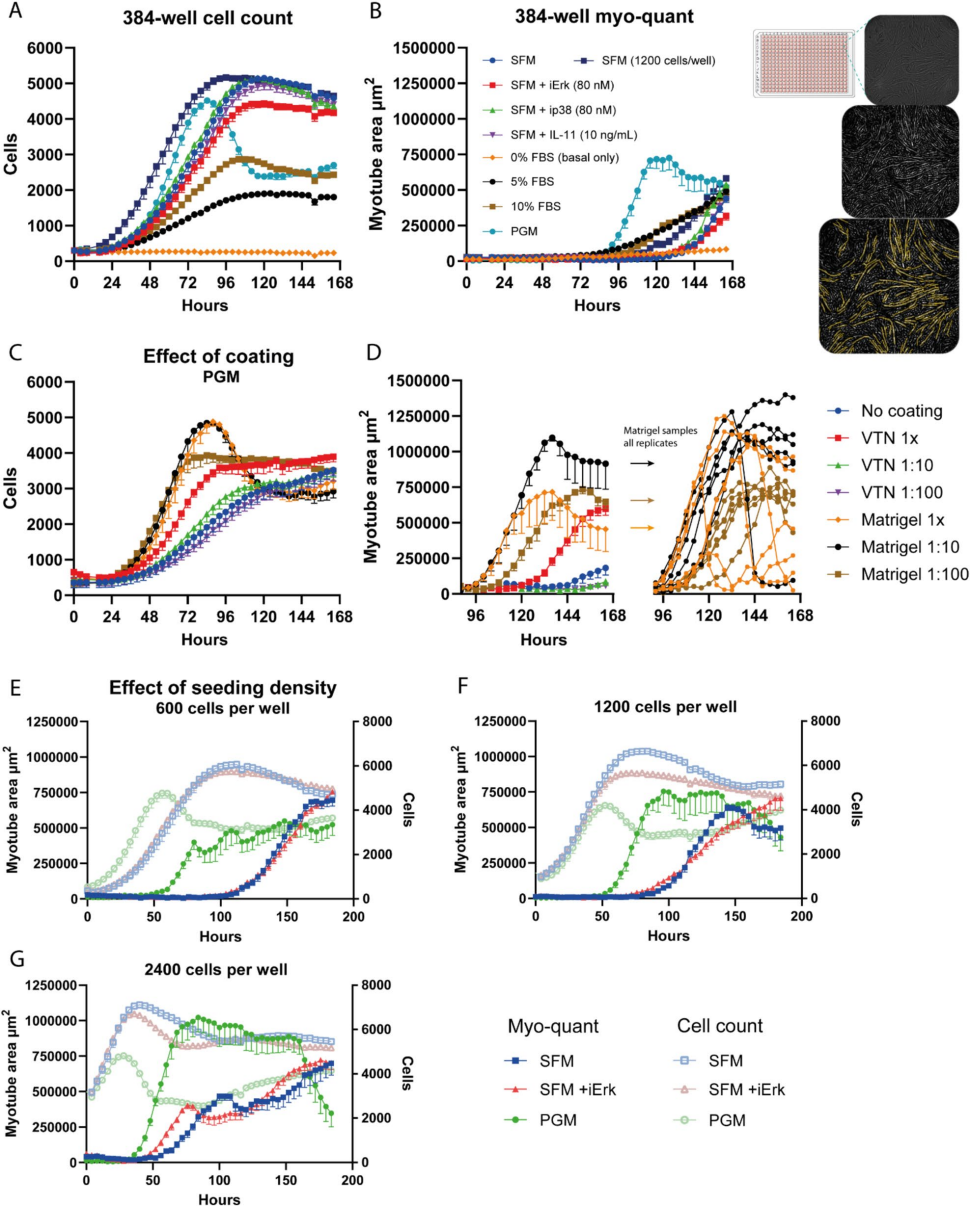
High-contrast brightfield (HCBF) imaging for myotube quantification in 384-well format. **A** + **B** Live assay HCBF cell count and myo-quant of BSCs with either 0%, 5% or 10% FBS, or with PGM or SFM with or without Erk inhibitor, p38 inhibitor or IL-11, all seeded at 600 cells/well. SFM was also measured with a 1200/well seeding density (*n* = 12–24). Representative images of a 384-well HCBF myo-quant are shown. **C** + **D** Live assay HCBF cell count and myo-quant analyses, as an effect of coating with either Matrigel or vitronectin (VTN) in 1x, 1:10 or 1:100 dilutions, or without any coating (*n* = 6). **E–G** Combined graph of HCBF cell count and myo-quant of BSCs seeded at either 600 cells/well (E), 1200 cells/well (F) or 2400 cells/well (G) and grown in SFM with or without Erk inhibitor (iErk, 80 nM) or PGM (*n* = 12). **A-D** is data from a VWR 384-well plate, while **E–G** data is from a Greiner 384-well plate. Data is presented as mean ± SEM

The HCBF myo-quant was then implemented and optimized for the 384-well format. Although we encountered several issues regarding the plate quality (discussed in Methods and Sup. Figure 7), we were able to perform a long-term live assay with simultaneous HCBF cell count and myo-quant with several different media types, all of them without any media change (Fig. 3A + B). Using the earlier established 600 cells/well seeding density, the HCBF cell count (Fig. 3A) shows the complete growth kinetics reaching a maximum plateau for all samples. There is also a very apparent drop in cell numbers, especially in the PGM group, signifying the start of differentiation. In terms of myotube formation (Fig. 3B), however, the timescale or seeding density did not seem to be sufficient to achieve maximum myotube area for all groups except the PGM which reach its plateau and subsequently drops after 120 h. Differentiation in SFM was initiated much earlier when 1200 cells/well instead of 600 cells/well was used as the initial seeding density (Fig. 3B), which is also reflected in the cell count data as this group reaches its maximum earlier than the other SFM groups (Fig. 3A). Interestingly, the addition of Erk inhibitor (iErk) significantly decreased the myo-quant by ~ 28% (p = 0.0069), while p38 inhibitor (ip38) increased it by ~ 22% (p = 0.0366) when comparing the data at the last time-point (166 h) (Sup. Figure 4 A). Due to the limited timescale and the quite unexpected negative effect of iErk, we further investigated this in a 96-well setup (Sup. Figure 4B + C). Here we observed a concentration-dependent effect of both inhibitors in the HCBF myo-quant assay, with the Erk inhibitor decreasing myo-kinetic growth initially yet achieving higher maximum myo-quant numbers with less myotube detachment at the end of the assay (~ 45% increase, p = 0.001), while the p38 inhibitor increased detachment after having an initial boost at the highest concentration. Therefore, Erk inhibition does seem to have a positive effect on myotube formation in long-term differentiation assays.

With the large number of available wells in the 384-well format we also tested the effect of coating on BSC proliferation and differentiation in the very same live assay experiment (Fig. 3C + D). The golden standard of Matrigel was compared with recombinant truncated vitronectin (VTN) both at three different concentrations. In PGM media, the cell growth was similar in Matrigel 1x and when diluted 1:10 (Fig. 3C), while the 1:10 diluted Matrigel surprisingly resulted in better myotube formation (Fig. 3D). Interestingly, the reason behind the better myo-quant numbers seemed to be due to a lesser extent of myotube detachment in the 1:10 dilution versus the 1 × samples, which can be more easily observed when showing all the replicates. Similarly, in SFM media, the Matrigel 1:10 samples also performed best in terms of both cell growth and myotube formation (Sup. Figure 5 A). In all cases, Matrigel was superior to VTN in the tested ranges where even 1:100 Matrigel dilution was at par with or better than VTN 1x, and VTN 1:10 and 1:100 seemed approximately at par with no coating. The coating also visibly affected the myotube morphology (Sup. Figure 5B).

Further investigations of BSC differentiation in SFM in the 384-well format were performed by increasing the initial seeding density to induce faster myo-kinetics (Fig. 3E-G). Both cell growth and myo-kinetics in SFM were affected by the increase in seeding density, with the initiation of myotube formation being approximately a day earlier going from 600 to 1200 cells/well, and one additional day earlier going from 1200 to 2400 cells/well, same as the plateau at maximum cell count numbers. Although the higher densities resulted in earlier initiation and peaks of the SFM differentiation, the maximally achievable myo-quant numbers did not change as the myotube detachment also increased at an earlier time-point, especially noticeable in the 2400 cells/well. The Erk inhibitor might have had a beneficial effect in reducing the myotube detachment at the 600 and 1200 cells/well seeding density, yet showing only a minor positive effect on SFM differentiation at the last time-point while significantly reducing the cell count (Sup. Figure 4D). All replicates for all the samples can be seen in Sup. Figure 4E for a better visualization of these detachment kinetics. PGM myotubes were less affected by the seeding in terms of their initiation, only being slightly faster in the highest cell density, however the maximum myo-quant numbers increased with higher cell densities. With this data it seems that the 1200 cells/well seeding density (Fig. 3F) might be the better choice for 384-well myo-quant, at least within this timeframe, considering both the PGM and SFM samples. However, an important note here is that this live assay was performed in another 384-well plate type (Greiner) than the first assay (VWR) in Fig. 3A-D. When comparing both the growth and myo-kinetics of BSCs grown under the same conditions it was apparent that the plate type (i.e. different manufacturers) could indeed affect both parameters (Sup. Figure 5 C). Therefore, all tested parameters (i.e. media type, seeding density, coating material and plate type) must be carefully considered when investigating growth and myo-kinetics.

### HCBF Imaging Highlights Cell Growth and Myo-kinetics of Porcine and First-Passage Bovine Satellite Cells

**Fig. 4:**
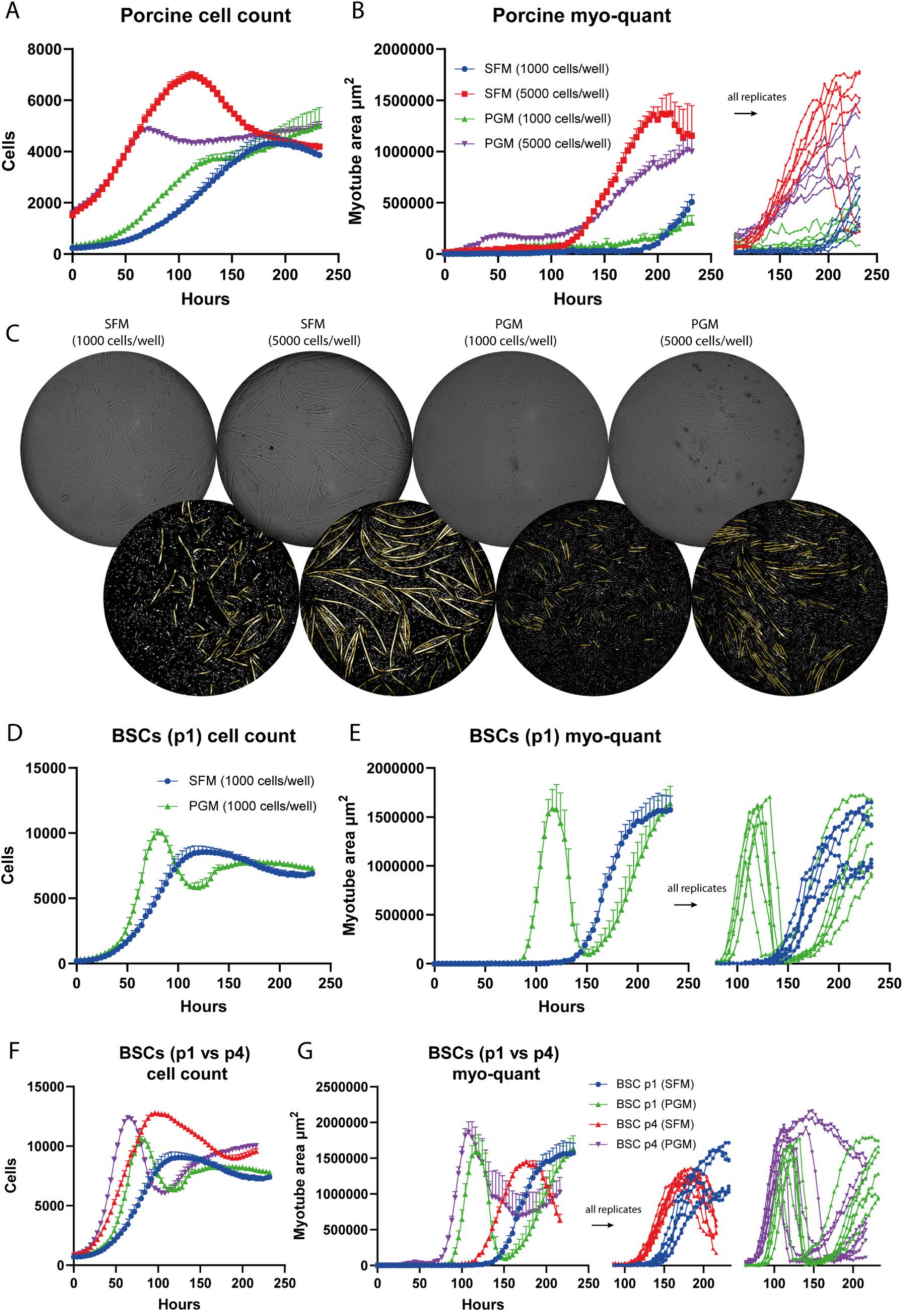
HCBF cell count and myo-quant of PSCs and early BSCs. **A** + **B** Live assay HCBF cell and myo-quant of PSCs seeded at either 1000 cells/well or 5000 cells/well and grown with SFM or PGM (without media change) in a 96-well format (*n* = 6). **C** Representative HCBF images and myo-quant analysis for the PSCs at the final time-point are shown. **D + E** Live assay HCBF cell and myo-quant of early first-passage (p1) bovine satellite cells (BSCs) seeded at 1000 cells/well and grown with SFM or PGM (without media change) in a 96-well format (*n* = 6). **F + G** Comparison between the HCBF live assay data from the p1 BSCs (D + E) and previously acquired data from p4 BSCs (Fig. 2) (*n* = 6–9). Data is presented as mean ± SEM

To assess the applicability of the HCBF cell count and myo-quant in another relevant species, we performed a long-term 96-well live assay with porcine satellite cells (PSCs) at two different seeding densities (1000 and 5000 cells/well) using either the SFM or PGM media (Fig. 4A-C). The PSCs were much slower growing than our BSCs, especially at the 1000 cells/well seeding density the PSCs reached their maximum cell numbers after nearly 200 h of cultivation with only minor myotube formation thereafter. However, increasing the initial seeding density to 5000 cells/well improved cell growth and myo-kinetics dramatically, indicating that the PSCs were even more sensitive to the effect of cell-density and crosstalk between cells than the BSCs (Fig. 1I + J). Surprisingly, with this seeding density the SFM showed a similar growth rate to the PGM, yet with a higher peak cell density (Fig. 4A) and with a much better differentiation (Fig. 4B). The morphology of the myotubes was also quite different between the two media types with much larger structures in the SFM (Fig. 4C).

Bovine satellite cells from the first passage after isolation were also analyzed in this live assay to ascertain the kinetic profile of these earlier and less activated cells (Fig. 4D + E). These BSCs grow well in both SFM and PGM, with higher growth rate and peak cell numbers in PGM which start to diverge and outperform the SFM after approximately 2 days (Fig. 4D). The PGM group has more than double the cell numbers of SFM at day 3 (*p* < 0.0001), while SFM has higher cell counts at day 5 (~ 46% higher, *p* = 0.0005) due to the differentiation induced drop with PGM (Sup. Figure 6 A). Surprisingly, the HCBF myo-quant showed a very characteristic differentiation in PGM with two clearly defined myotube peaks at a similar maximum surface area although with much slower myo-kinetics in the second peak (Fig. 4E). When showing all replicates it is evident that they all go through complete myotube detachment followed by an effective reforming of the secondary myotubes which appeared with a very different myotube morphology probably due to the already dense cell population and limited surface area (Sup. Figure 6B). Differentiation in SFM was also successful with similar maximum myo-quant numbers as the PGM, albeit at a slower rate and with a delay of more than 100 h (Fig. 4E, Sup. Figure 6 A). Furthermore, when this first-passage (p1) BSC data was compared to the earlier live assay data (Fig. 2), which was from BSCs passaged four times (p4) from the same donor, it was observed that the p1 BSCs had both slower cell growth and differentiation (Fig. 4F + G). In the PGM this was mainly due to an initial delay in both the exponential growth phase and the differentiation phase, while for SFM it was both a delay and a slower growth rate with lower maximum cell density before differentiation also showing higher variation between replicates (Sup. Figure 6 C + D). However, the p1 BSCs in SFM did not show any myotube detachment within the timeframe of the live assay and therefore achieved higher final myo-quant numbers compared to the p4 BSCs (Fig. 4G).

### High-throughput Label-Free Screening of Clonal Satellite Cell Populations

**Fig. 5:**
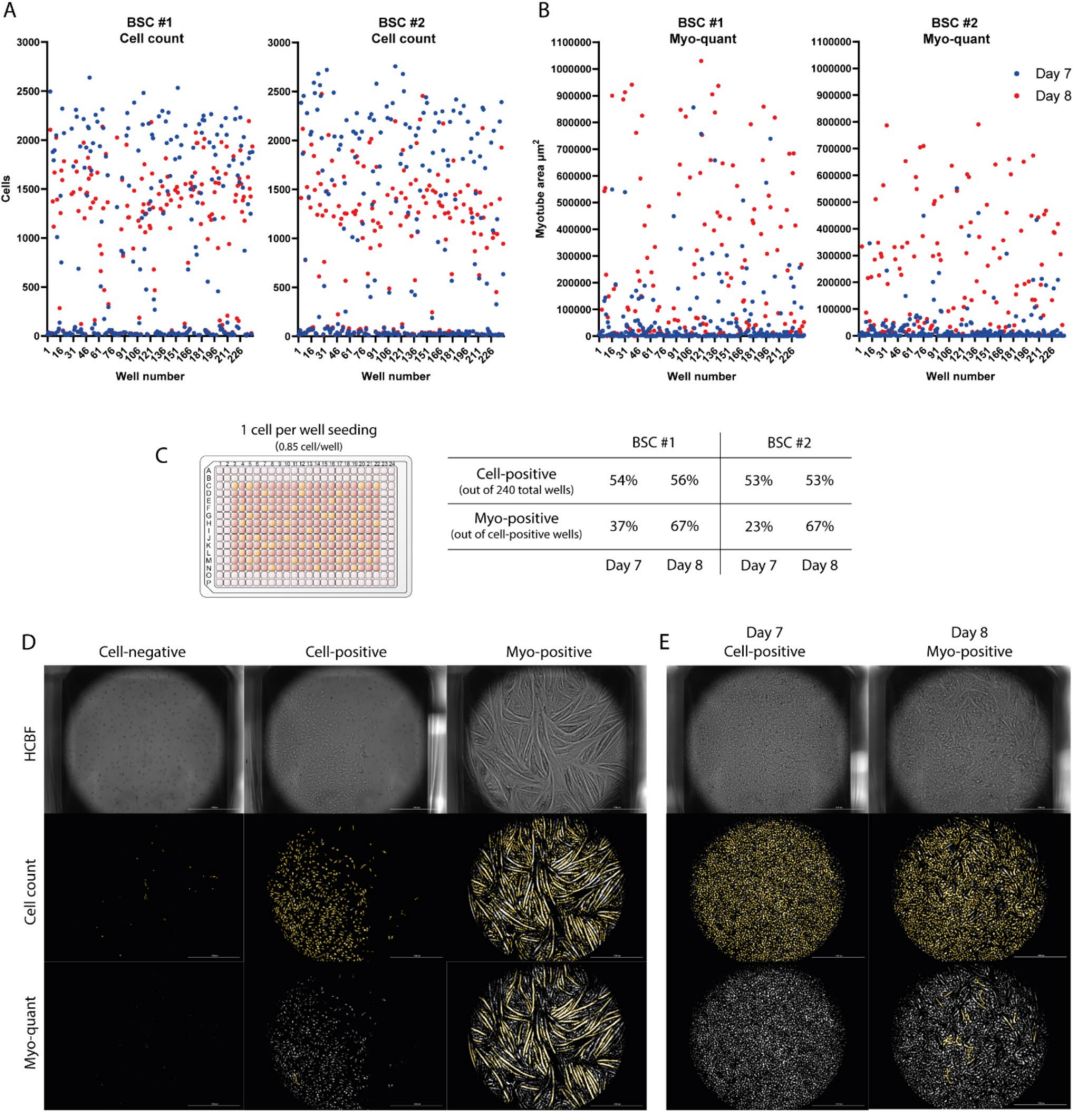
HCBF cell count and myo-quant screening of early first-passage BSCs in a 384-well format. **A** + **B** HCBF cell count and myo-quant of the 240 tested wells on day 7 and day 8 after seeding 0.85 cells/well in PGM media using p1 BSCs from two different donors (#1 and #2). The experiment was performed in a VWR 384-well plate without any media change. **C** The percentage of cell-positive wells (out of the total 240 wells) and myo-positive wells (out of the cell-positive wells). The cell-positive threshold was 120 cells to avoid debris and plate artefacts being falsely counted as positive wells. The myo-positive threshold was 60.000 µm^2^ to avoid plate artefacts and dense non-myogenic cell clusters being falsely counted as positive wells. **D** Representative HCBF cell count of myo-quant images showing a cell-negative, cell-positive and myo-positive well after 8 days. **E** Representative HCBF cell count of myo-quant images showing the same well at day 7 and day 8 as it become myo-positive

To further explore the applicability of the high-throughput HCBF cell count and myo-quant we examined if the early first-passage bovine satellite cells had the ability to grow and differentiate starting with only one single cell per well. If the resulting clonal culture would be successful in forming myotubes, it would mean that the single starting cell could only be of proper satellite stem cell origin. To test this, we used an initial seeding density of 0.85 cells/well in the 384-well plate format using PGM and performed HCBF cell counting and myo-quant after 7 and 8 days of cultivation (Fig. 5A + B). This setup was performed on BSCs (p1) from two different donors (BSC #1 and BSC #2) using one 384-well plate per donor. Instead of using two different HCBF imaging channels and defocus settings (as in all the previous results), we only used the extra defocused myo-quant setting for both the HCBF cell count and myo-quant analysis. This slightly reduced the precision of the cell count, yet it was still robust enough to identify cell-positive and cell-negative wells while reducing the imaging time, data handling and file size. The percentage of cell-positive wells was quite low and only changed from 54 to 56% in the BSC #1, going from day 7 to day 8, while remaining at 53% in the BSC #2 (Fig. 5A + B). The myo-positive wells, however, increased dramatically from 37 to 67% and from 23 to 67%, in the BSC #1 and #2 respectively (Fig. 5B + C). This change from day 7 to day 8 in the amount of differentiation is clearly visible in the myo-quant graph (Fig. 5B), yet it should also be noted that it correlates with a decrease in cell numbers (Fig. 5A) which are to be expected given the previous live assay results. Remarkably, some clonal BSC populations were able to completely cover the entire well with large myotubes after only 8 days starting from the one single cell (Fig. 5D), while other populations were not even close to 100% confluence. While some of these cell-positive wells without myotubes might be non-myogenic populations, there might also be very slow growing and differentiating satellite cell populations, as several wells became myo-positive only on day 8 (Fig. 5E). This could imply that extending the HCBF imaging further to include additional cultivation days would result in higher percentages of myo-positive wells. Nevertheless, this still highlights the very different growth and differentiation characteristics of the clonal satellite cell populations and thereby the heterogeneity in the original isolated population.

## Discussion

In this study, we introduced a novel high-contrast brightfield (HCBF) imaging technique for quantitative assessment of both proliferation and myotube formation in a high-throughput format, offering a dynamic label-free approach applicable to cultivated meat and muscle cell research. When compared to traditional end-point fluorescence imaging of stained samples, the HCBF imaging offers several advantages such as highly time-resolved kinetic data with much less labor and samples needed, no additional reagents needed, no myotube detachment caused by staining/washing procedures and the ability to ascertain both proliferation and differentiation data in one single assay. With this technique we highlighted the very dynamic behavior of satellite cells undergoing myogenic differentiation and the difficulty in quantifying the myotubes due to their sporadic formation and detachment.

In this study, we utilized the high-throughput and detailed kinetic data acquisition of the HCBF imaging to optimize several cell culture parameters. The formation of myotubes in our in-house developed and well-characterized serum-free media[12, 17, 31], which was much delayed compared with serum-containing media, was found to be improved by simply leaving the media throughout the entire live cell assay without any media change with reduced components. This approach also worked well with the serum-containing media. These results could be explained by the accumulation of secreted growth factors and metabolites from the SCs which in turn also consumes the macronutrients while lowering the pH, mainly due to lactate production, potentially resulting in a more natural state of stress-induced myogenic differentiation [32–35]. Surprisingly, we found that the formation of porcine myotubes was significantly better when using our serum-free media compared to the serum-containing media, which suggests that there are species-specific media requirements to be considered for optimized myogenic differentiation. We have also previously reported the positive effect of our serum-free media in the accumulation of lipid droplets within porcine satellite cells [30].

The minor positive effect we observed with Erk inhibitor addition seemed to be due to lower myotube detachment ultimately leading to higher myo-quant numbers at later timepoints, while it initially reduced the myo-kinetics, although we might have had higher efficacies using higher concentrations reported by other studies [36, 37]. We also discovered that a further 1:10 dilution of Matrigel improved myotube formation, an effect mainly attributed to the reduced myotube detachment, which highlights the importance of properly controlled mechanical stimuli and cell-substrate binding interactions [38, 39]. In the same manner, the mechanical properties of the two types of 384-well plates (Greiner and VWR) were likely the cause for the differing growth and myo-kinetics that we observed. Basic cell culture parameters, such as seeding density, were established for the 384-well format both regarding proper growth and differentiation within an acceptable timeframe. The plate quality issues we experienced with the 384-well formats were unfortunate with similar optical issues being reported by others [26], though we expect there might be a better product for exactly this type of HCBF imaging. Despite the quality issues, the very high-throughput format of the 384-well plates allowed for many parameters to be efficiently tested with high replicate numbers within one single assay. As a result of this high-throughput format the 384-well plate also enables an efficient experimental setup to screen the overall myogenicity of the starting cell population and the performance of individual clonal satellite cell populations. Furthermore, as with the nature of the label-free imaging, this also allows for a clonal selection process in which the better performing satellite cells could be further cultivated and characterized based on their growth and myogenicity.

The HCBF myo-quant of the bovine satellite cells, especially the early first-passage cells, highlighted a very interesting cellular phenomena showing first a myotube detachment and then a reformation with a second wave of morphologically different myotubes. This exact phenomenon was also reported recently by Melzener and colleagues [37], in which they also characterized the initially non-fused satellite cells using single-cell RNA-seq as a pool of reserve cells that retained their ability to undergo myogenic differentiation. The secondary myotubes would indeed also be interesting to characterize in future studies to understand if the changed morphology is only based on the limitation of surface area or if there are any differential expression of key myogenic markers [40].

There are of course limitations to be considered when using HCBF imaging. As quantification is based on contrast created by the cells themselves with visible light, it is important that both the samples (e.g. cell morphology, cell debris and media color) and plasticware (e.g. clarity and scratches) do not introduce light artefacts or variations. This was especially apparent with the quality issues we encountered with the 384-well plates. The HCBF imaging technique is not as sensitive as fluorescence-enabled imaging, not as detailed in terms of myotube morphology visualization and does not allow for fusion index determination. Especially, the more complex and spread-out myotubes formed under serum conditions (PGM) were difficult to precisely quantify. HCBF cell count was very robust up until fully confluent cultures, yet it cannot quantify cells after differentiation like the Hoechst-stained nuclei counting. Furthermore, fluorescence imaging also enables the imaging of the entire well, without noticeable meniscus-induced light effects along the edges, ultimately increasing data collection and can also give additional information about protein expression. Yet, the end-point nature of laboriously fixing and staining samples, using expensive and toxic reagents, with the added risk of mechanically disturbing and detaching myotubes [7], instill some major limitations with labelling methods in terms of high-throughput and kinetic data acquisition. It is however possible to quantify myotubes in real-time with fluorescence imaging, albeit this requires fluorescent protein-labelling via genetic engineering [7, 36, 41]. While genetically engineered cells expressing fluorescent proteins provide detailed insights into muscle cell differentiation, the generation and validation of these can be challenging and time-consuming [42]. Furthermore, it may not reflect the real behavior of unedited satellite cells as labelling has been shown to alter the protein dynamics [43], while vector transfection causes several stresses, off-targets and other artefacts [44]. There is also the added complication of phototoxicity when live cells are continuously exposed to fluorescence imaging with intracellular fluorescent proteins [45]. Moreover, this editing can only be feasibly implemented in immortalized cell lines and not primary satellite cells perhaps with the exception of murine cells generated by transgene editing and breeding [36]. These many challenges and considerations are ultimately solved by HCBF imaging, which can be used with normal primary satellite cells without any additional work, while using common inexpensive culture plates contrary to the impedance-based techniques (e.g. xCELLigence) which require specialized microplates with gold microelectrode biosensors [46].

Our work was centered around the use of the BioTek Cytation 5 and the well-integrated Gen5 software from Agilent Technologies, yet open-source myotube analyzers do exist, like Myotube Analyzer [47], MyoCount [48] and ImageJ macros [19] for those without the availability of powerful equipment/software packages such as the Cytation 5/Gen5. Herein also lies several additional parameters to quantify e.g. nuclei clustering and myotube branching [47] or myotube width and nuclear/cytoplasmic ratios [19], which might be relevant to answer some specific pathological research questions. Here it might also be interesting to consider the possibility of improving HCBF myo-quant analysis by third-party software suites or by using AI-powered tools with the ability to recognize patterns and shapes instead of solely relying on the contrast generated by the myotubes through HCBF imaging [14–16]. Such an label-free approach was even achieved for complex 3D organoids in a recent study [49], yet requires large annotated datasets for neural network training. In terms of high-throughput cell imaging, other imaging systems like Spark Cyto (Tecan), Omni (Axion Biosystems), Incucyte (Sartorius) and several more promise label-free cell growth monitoring through brightfield imaging (though not high-contrast), however whether these systems can also be tweaked to work with myotube quantification remains to be seen. Nevertheless, the contrast of normal brightfield imaging can be increased by different defocusing strategies [24–26] and has previously been greatly enhanced by cleverly combining a self-constructed pinhole, color filter, defocusing and PBS-induced cell swelling [28]. Label-free myotube quantification will therefore likely be achievable with other microscope systems in combination with powerful open-source image analyzers, perhaps further enhanced by trained deep learning models [50], although with a more complicated workflow compared with well-integrated automated imager/software packages such as the BioTek Cytation 5 system. A key strength with our methodology is that we simply use the existing features developed for cell counting with tweaked parameters, making it easy to implement for other Cytation users, albeit with obvious limitations in its applicability for researchers without similar imager platforms. Ultimately, we hope that our HCBF myo-quant approach inspires academic groups as well as companies to investigate and implement similar strategies for improved image-based cytometry of muscle stem cells undergoing myogenic differentiation.

While the HCBF cell count and myo-quant was tested using bovine and porcine satellite cells, the technique would likely also work with human satellite cells and could possibly have exciting applications in the research areas of human muscle pathology, musculoskeletal-focused drug discovery, skeletal muscle tissue engineering and in our understanding of myogenesis in general. Furthermore, the myotube detachment kinetics identified in our study would be interesting to investigate further, e.g. with acetylcholine stimulation, as it might depend on contractibility [51] and could therefore have major implications for additional applications within neuromuscular pathophysiological screening.

## Conclusion

HCBF imaging enables robust label-free quantification of cell proliferation and myogenic differentiation of muscle stem cells in both 96- and 384-well formats. First and foremost, HCBF-based myotube quantification should be seen as an efficient low cost, high-throughput screening tool for myotube formation with no additional cell work needed. However, where this technique adds to our understanding, is when HCBF is used in a high-throughput high-resolution live assay setting in which the complete proliferation and myo-kinetics can be ascertained, as well as the rate of myotube detachment. The full dynamic profile of primary satellite cells from single cells to large myotubes in one single assay, without the need for gene-editing or any other labelling strategy or even specially designed microplates, is a major step towards better and more efficient muscle stem cell research and serum-free media optimization for cultivated meat.

## Supplementary Information

Below is the link to the electronic supplementary material.

Supplementary file1 (DOCX 9697 KB)

Video 1: Live assay with continuous HCBF cell count and myo-quant with detaching and reforming myotubes. Images from the HCBF cell count (left), HCBF imaging (cell count defocus setting, middle) and HCBF myo-quant (right) are shown side-by-side for one PGM well replicate in which myotubes are detaching and reforming (also highlighted in Fig 2. F+G). HCBF imaging here in 96-well format is performed in 4-hour intervals. Supplementary file2 (MOV 793 MB)

## Declarations

### Data Availability

Additional data, raw data, videos and images of cells are available upon request.

## References

[1] Sinke, P., Swartz, E., Sanctorum, H., van der Giesen, C., & Odegard, I. (2023). Ex-ante life cycle assessment of commercial-scale cultivated meat production in 2030. *International Journal of Life Cycle Assessment,**28*, 234–254. 10.1007/s11367-022-02128-8

[2] Post, M. J., Levenberg, S., Kaplan, D. L., Genovese, N., Fu, J., Bryant, C. J., Negowetti, N., Verzijden, K., & Moutsatsou, P. (2020). Scientific, sustainability and regulatory challenges of cultured meat. *Nature Food,**1*, 403–415.

[3] Rasmussen, M. K., Gold, J., Kaiser, M. W., Moritz, J., Räty, N., Rønning, S. B., Ryynänen, T., Skrivergaard, S., Ström, A., Therkildsen, M., et al. (2024). Critical review of cultivated meat from a Nordic perspective. *Trends in Food Science & Technology,**144*, 104336. 10.1016/J.TIFS.2024.104336

[4] Juhas, M., Ye, J., & Bursac, N. (2016). Design, evaluation, and application of engineered skeletal muscle. *Methods,**99*, 81–90. 10.1016/J.YMETH.2015.10.002 26455485 10.1016/j.ymeth.2015.10.002 PMC4821818

[5] Yin, H., Price, F., & Rudnicki, M. A. (2013). Satellite cells and the muscle stem cell niche. *Physiological Reviews,**93*, 23–67. 10.1152/physrev.00043.2011 23303905 10.1152/physrev.00043.2011 PMC4073943

[6] Benam, K. H., Dauth, S., Hassell, B., Herland, A., Jain, A., Jang, K. J., Karalis, K., Kim, H. J., MacQueen, L., Mahmoodian, R., et al. (2015). Engineered in vitro disease models. *Annual Review of Pathology: Mechanisms of Disease,**10*, 195–262. 10.1146/ANNUREV-PATHOL-012414-040418/CITE/REFWORKS 10.1146/annurev-pathol-012414-040418 25621660

[7] Sapoznik, E., Niu, G., Zhou, Y., Prim, P. M., Criswell, T. L., & Soker, S. (2018). A real-time monitoring platform of myogenesis regulators using double fluorescent labeling. *PLoS ONE,**13*, e0192654. 10.1371/JOURNAL.PONE.0192654 29444187 10.1371/journal.pone.0192654 PMC5812636

[8] Specht, E. A., Welch, D. R., Rees Clayton, E. M., & Lagally, C. D. (2018). Opportunities for applying biomedical production and manufacturing methods to the development of the clean meat industry. *Biochemical Engineering Journal,**132*, 161–168. 10.1016/j.bej.2018.01.015

[9] Messmer, T., Klevernic, I., Furquim, C., Ovchinnikova, E., Dogan, A., Cruz, H., Post, M. J., & Flack, J. E. (2022). A serum-free media formulation for cultured meat production supports bovine satellite cell differentiation in the absence of serum starvation. *Nature Food,**3*, 74–85. 10.1038/s43016-021-00419-1 37118488 10.1038/s43016-021-00419-1

[10] Zuncheddu, D., Della Bella, E., Schwab, A., Petta, D., Rocchitta, G., Generelli, S., Kurth, F., Parrilli, A., Verrier, S., Rau, J. V., et al. (2021). Quality control methods in musculoskeletal tissue engineering: From imaging to biosensors. *Bone Research.,**9*, 1–21. 10.1038/s41413-021-00167-9 34707086 10.1038/s41413-021-00167-9 PMC8551153

[11] Pegoraro, G., & Misteli, T. (2017). High-throughput imaging for the discovery of cellular mechanisms of disease. *Trends in Genetics,**33*, 604. 10.1016/J.TIG.2017.06.005 28732598 10.1016/j.tig.2017.06.005 PMC5562530

[12] Skrivergaard, S., Young, J. F., Sahebekhtiari, N., Semper, C., Venkatesan, M., Savchenko, A., Stogios, P. J., Therkildsen, M., & Rasmussen, M. K. (2023). A simple and robust serum-free media for the proliferation of muscle cells. *Food Research International,**172*, 1–54. 10.1016/j.foodres.2023.113194 10.1016/j.foodres.2023.113194 37689947

[13] Robinson, J. P. (2005). Comparative overview of flow and image cytometry. *Current Protocols in Cytometry,**31*, 1211–12111. 10.1002/0471142956.CY1201S31 10.1002/0471142956.cy1201s31 18770812

[14] Bilodeau, A., Bouchard, C., & Lavoie-Cardinal, F. (2022). Automated microscopy image segmentation and analysis with machine learning. *Methods in Molecular Biology,**2440*, 349–365. 10.1007/978-1-0716-2051-9_20/FIGURES/4 35218549 10.1007/978-1-0716-2051-9_20

[15] McQuin, C., Goodman, A., Chernyshev, V., Kamentsky, L., Cimini, B. A., Karhohs, K. W., Doan, M., Ding, L., Rafelski, S. M., Thirstrup, D. et al. (2018). CellProfiler 3.0: Next-generation image processing for biology. *PLoS Biol*, *16*, 10.1371/JOURNAL.PBIO.2005970 10.1371/journal.pbio.2005970 PMC6029841 29969450

[16] Maddalena, L., Antonelli, L., Albu, A., Hada, A., & Guarracino, M. R. (2022). Artificial intelligence for cell segmentation, event detection, and tracking for label-free microscopy imaging. *Algorithms,**15*, 313. 10.3390/A15090313

[17] Skrivergaard, S., Krøyer Rasmussen, M., Sahebekhtiari, N., Feveile Young, J., & Therkildsen, M. (2023). Satellite cells sourced from bull calves and dairy cows differs in proliferative and myogenic capacity– Implications for cultivated meat. *Food Research International,**173*, 113217. 10.1016/j.foodres.2023.113217 37803537 10.1016/j.foodres.2023.113217

[18] Melzener, L., Ding, S., Hueber, R., Messmer, T., Zhou, G., Post, M. J., Flack, J. E. (2022). Comparative analysis of cattle breeds as satellite cell donors for cultured beef. *bioRxiv*, 2022.01.14.476358, 10.1101/2022.01.14.476358.

[19] Jurberg, A. D., Gomes, G., Seixas, M. R., Mermelstein, C., & Costa, M. L. (2023). Improving quantification of myotube width and nuclear/cytoplasmic ratio in myogenesis research. *Computer Methods and Programs in Biomedicine,**230*, 107354. 10.1016/J.CMPB.2023.107354 36682109 10.1016/j.cmpb.2023.107354

[20] Park, I., Hong, Y., Jun, Y. H., Lee, G. Y., Jun, H. S., Pyun, J. C., Choi, J. W., & Cho, S. (2016). Electrical impedance monitoring of C2C12 myoblast differentiation on an indium tin oxide electrode. *Sensors,**16*, 2068. 10.3390/S16122068 27929401 10.3390/s16122068 PMC5191049

[21] Ikeda, K., Ito, A., Imada, R., Sato, M., Kawabe, Y., & Kamihira, M. (2017). In vitro drug testing based on contractile activity of C2C12 cells in an epigenetic drug model. *Scientific reports,**7*, 1–11. 10.1038/srep44570 28300163 10.1038/srep44570 PMC5353687

[22] Vandenburgh, H., Shansky, J., Benesch-Lee, F., Barbata, V., Reid, J., Thorrez, L., Valentini, R., & Crawford, G. (2008). Drug-screening platform based on the contractility of tissue-engineered muscle. *Muscle and Nerve,**37*, 438–447. 10.1002/MUS.20931 18236465 10.1002/mus.20931

[23] Chen, B., Yin, Z., Ng, B.W.-L., Wang, D. M., Tuan, R. S., Bise, R., & Ker, D. F. E. (2024). Label-free live cell recognition and tracking for biological discoveries and translational applications. *npj Imaging,**2*, 1–26. 10.1038/s44303-024-00046-y 40603709 10.1038/s44303-024-00046-y PMC12118707

[24] Agero, U., Monken, C. H., Ropert, C., Gazzinelli, R. T., & Mesquita, O. N. (2003). Cell surface fluctuations studied with defocusing microscopy. *Physical Review E,**67*, 9. 10.1103/PHYSREVE.67.051904/FIGURE/1/THUMB 10.1103/PhysRevE.67.051904 12786175

[25] Selinummi, J., Ruusuvuori, P., Podolsky, I., Ozinsky, A., Gold, E., Yli-Harja, O., Aderem, A., & Shmulevich, I. (2009). Bright field microscopy as an alternative to whole cell fluorescence in automated analysis of macrophage images. *PLoS ONE,**4*, e7497. 10.1371/JOURNAL.PONE.0007497 19847301 10.1371/journal.pone.0007497 PMC2760782

[26] Dehlinger, D., Suer, L., Elsheikh, M., Peña, J., & Naraghi-Arani, P. (2013). Dye free automated cell counting and analysis. *Biotechnology and Bioengineering,**110*, 838–847. 10.1002/BIT.24757 23055412 10.1002/bit.24757

[27] Carlsen, J., Cömert, C., Bross, P., Palmfeldt, J. (2020). Optimized high-contrast brightfield microscopy application for noninvasive proliferation assays of human cell cultures. *18*, 215–225. https://home.liebertpub.com/adt. 10.1089/ADT.2020.981 10.1089/adt.2020.981 32692633

[28] Drey, L. L., Graber, M. C., & Bieschke, J. (2013). Counting unstained, confluent cells by modified bright-field microscopy. *BioTechniques,**55*, 28–33. 10.2144/000114056/ASSET/IMAGES/LARGE/FIGURE4.JPEG 23834382 10.2144/000114056 PMC3864689

[29] Skrivergaard, S., Rasmussen, M. K., Therkildsen, M., Young, J. F. (2021). Bovine satellite cells isolated after 2 and 5 days of tissue storage maintain the proliferative and myogenic capacity needed for cultured meat production. *International Journal of Molecular Sciences*, *22*, 8376. 10.3390/IJMS22168376 10.3390/ijms22168376 PMC8395070 34445082

[30] Abraham, A., Auguet-Lara, M., Skrivergaard, S., Therkildsen, M., Rasmussen, M. K., Young, J. F. (2024). Reduced and serum-free media facilitates lipid accumulation in porcine satellite cells; Possible implications for cultivated meat. 10.2139/SSRN.4872912

[31] El Wali, M., Karinen, H., Rønning, S. B., Skrivergaard, S., Dorca-Preda, T., Rasmussen, M. K., Young, J. F., Therkildsen, M., Mogensen, L., Ryynänen, T., et al. (2024). Life cycle assessment of culture media with alternative compositions for cultured meat production. *The International Journal of Life Cycle Assessment,**29*, 2077–2093. 10.1007/S11367-024-02350-6/TABLES/3

[32] Seo, E., Kang, H., Lim, O. K., Jun, H. S. (2018). Supplementation with IL-6 and muscle cell culture conditioned media enhances myogenic differentiation of adipose tissue-derived stem cells through STAT3 activation. *International Journal of Molecular Sciences*, *19*, 1557, 10.3390/IJMS19061557 10.3390/ijms19061557 PMC6032255 29882916

[33] Kim, M. J., Kim, Z. H., Kim, S. M., & Choi, Y. S. (2016). Conditioned medium derived from umbilical cord mesenchymal stem cells regenerates atrophied muscles. *Tissue and Cell,**48*, 533–543. 10.1016/J.TICE.2016.06.010 27457384 10.1016/j.tice.2016.06.010

[34] Tsukamoto, S., Shibasaki, A., Naka, A., Saito, H., Iida, K. (2018). Lactate promotes myoblast differentiation and myotube hypertrophy via a pathway involving MyoD in vitro and enhances muscle regeneration in vivo. *International Journal of Molecular Sciences*, *19*. 10.3390/ijms19113649 10.3390/ijms19113649 PMC6274869 30463265

[35] Nielsen, S. D. H., Sahebekhtiari, N., Huang, Z., Young, J. F., & Rasmussen, M. K. (2024). Comparison of secreted miRNAs and proteins during proliferation and differentiation of bovine satellite cells in culture implies potential roles in regulating myogenesis. *Gene,**894*, 147979. 10.1016/J.GENE.2023.147979 37952749 10.1016/j.gene.2023.147979

[36] Eigler, T., Zarfati, G., Amzallag, E., Sinha, S., Segev, N., Zabary, Y., Zaritsky, A., Shakked, A., Umansky, K. B., Schejter, E. D., et al. (2021). ERK1/2 inhibition promotes robust myotube growth via CaMKII activation resulting in myoblast-to-myotube fusion. *Developmental Cell,**56*, 3349-3363.e6. 10.1016/J.DEVCEL.2021.11.022 34932950 10.1016/j.devcel.2021.11.022 PMC8693863

[37] Melzener, L., Schaeken, L., Fros, M., Messmer, T., Raina, D., Kiessling, A., van Haaften, T., Spaans, S., Doǧan, A., Post, M. J., et al. (2024). Optimisation of cell fate determination for cultivated muscle differentiation. *Communications Biology,**7*, 1–12. 10.1038/s42003-024-07201-6 39532984 10.1038/s42003-024-07201-6 PMC11557827

[38] Engler, A. J., Griffin, M. A., Sen, S., Bönnemann, C. G., Sweeney, H. L., & Discher, D. E. (2004). Myotubes differentiate optimally on substrates with tissue-like stiffness: Pathological implications for soft or stiff microenvironments. *Journal of Cell Biology,**166*, 877–887. 10.1083/jcb.200405004 15364962 10.1083/jcb.200405004 PMC2172122

[39] Lin, X., Shi, Y., Cao, Y., & Liu, W. (2016). Recent progress in stem cell differentiation directed by material and mechanical cues. *Biomedical Materials,**11*, 014109. 10.1088/1748-6041/11/1/014109 26836059 10.1088/1748-6041/11/1/014109

[40] Auguet-Lara, M., Skrivergaard, S., Therkildsen, M., Rasmussen, M. K., & Young, J. F. (2025). Development of a biomarker panel for cell characterization intended for cultivated meat. *Experimental Cell Research,**446*, 114467. 10.1016/J.YEXCR.2025.114467 39978714 10.1016/j.yexcr.2025.114467

[41] Kodaka, M., Yang, Z., Nakagawa, K., Maruyama, J., Xu, X., Sarkar, A., Ichimura, A., Nasu, Y., Ozawa, T., Iwasa, H., et al. (2015). A new cell-based assay to evaluate myogenesis in mouse myoblast C2C12 cells. *Experimental Cell Research,**336*, 171–181. 10.1016/J.YEXCR.2015.06.015 26116467 10.1016/j.yexcr.2015.06.015

[42] Koch, B., Nijmeijer, B., Kueblbeck, M., Cai, Y., Walther, N., & Ellenberg, J. (2018). Generation and validation of homozygous fluorescent knock-in cells using CRISPR/Cas9 genome editing. *Nature Protocols,**13*, 1465. 10.1038/NPROT.2018.042 29844520 10.1038/nprot.2018.042 PMC6556379

[43] Yin, L., Wang, W., Wang, S., Zhang, F., Zhang, S., & Tao, N. (2015). How does fluorescent labeling affect the binding kinetics of proteins with intact cells? *Biosensors & Bioelectronics,**66*, 412. 10.1016/J.BIOS.2014.11.036 25486538 10.1016/j.bios.2014.11.036 PMC4836836

[44] Stepanenko, A. A., & Heng, H. H. (2017). Transient and stable vector transfection: Pitfalls, off-target effects, artifacts. *Mutation Research/Reviews in Mutation Research,**773*, 91–103. 10.1016/J.MRREV.2017.05.002 28927539 10.1016/j.mrrev.2017.05.002

[45] Laissue, P. P., Alghamdi, R. A., Tomancak, P., Reynaud, E. G., & Shroff, H. (2017). Assessing phototoxicity in live fluorescence imaging. *Nature Methods,**14*, 657–661. 10.1038/nmeth.4344 28661494 10.1038/nmeth.4344

[46] Murphy, S. M., Kiely, M., Jakeman, P. M., Kiely, P. A., & Carson, B. P. (2016). Optimization of an in vitro bioassay to monitor growth and formation of myotubes in real time. *Bioscience Reports,**36*, e00330. 10.1042/BSR20160036 27009307 10.1042/BSR20160036 PMC4859084

[47] Noë, S., Corvelyn, M., Willems, S., Costamagna, D., Aerts, J. M., Van Campenhout, A., & Desloovere, K. (2022). The Myotube Analyzer: How to assess myogenic features in muscle stem cells. *Skeletal Muscle,**12*, 1–12. 10.1186/S13395-022-00297-6/FIGURES/10 35689270 10.1186/s13395-022-00297-6 PMC9185954

[48] Murphy, D. P., Nicholson, T., Jones, S. W., & O’Leary, M. F. (2019). MyoCount: A software tool for the automated quantification of myotube surface area and nuclear fusion index [version 1; referees: 2 approved]. *Wellcome Open Research,**4*, 6. 10.12688/wellcomeopenres.15055.1 30906880 10.12688/wellcomeopenres.15055.1 PMC6419977

[49] Kok, R. N. U., Spoelstra, W. K., Betjes, M. A., van Zon, J. S., & Tans, S. J. (2025). Label-free cell imaging and tracking in 3D organoids. *Cell Reports Physical Science,**6*, 102522. 10.1016/J.XCRP.2025.102522

[50] Cross-Zamirski, J. O., Mouchet, E., Williams, G., Schönlieb, C. B., Turkki, R., & Wang, Y. (2022). Label-free prediction of cell painting from brightfield images. *Scientific Reports,**12*, 1–13. 10.1038/s41598-022-12914-x 35705591 10.1038/s41598-022-12914-x PMC9200748

[51] Young, J., Margaron, Y., Fernandes, M., Duchemin-Pelletier, E., Michaud, J., Flaender, M., Lorintiu, O., Degot, S., & Poydenot, P. (2018). MyoScreen, a high-throughput phenotypic screening platform enabling muscle drug discovery. *SLAS Discovery,**23*, 790–806. 10.1177/2472555218761102 29498891 10.1177/2472555218761102

