# Supplementary material for: High-Throughput Label-Free Continuous Quantification of Muscle Stem Cell Proliferation and Myogenic Differentiation
Source: Stem Cell Rev Rep. 2025 Jul 3;21(7):2103–20. doi: 10.1007/s12015-025-10915-7 (PMC12408787; doi:10.1007/s12015-025-10915-7)
Supplement: Supplementary file 1 — Supplementary file1 (DOCX 9697 KB) [file 12015_2025_10915_MOESM1_ESM.docx]

**Supplementary Data**


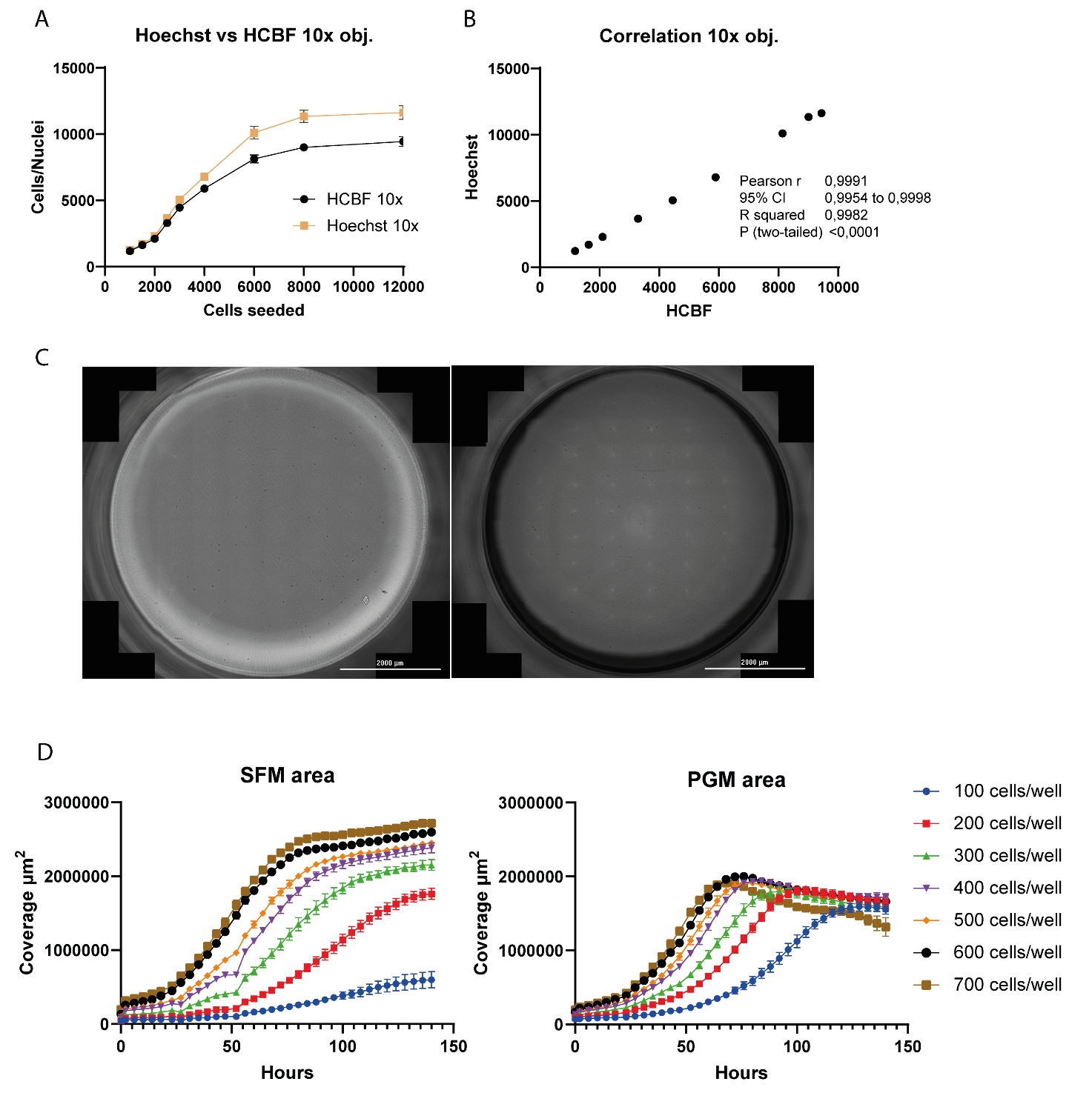


**Supplementary Figure 1:
A+B** Comparison and correlation between Hoechst-stained nuclei counting and HCBF cell counting using the 10x objective (instead of 4x) in a 96-well format (n = 4). **C** Representative HCBF imaging showing very little meniscus effect in 96-well format when using the 10x objective even with low well liquid volume (30 µL) (right image) when compared to a well without liquid (left image). **D** Live assay graphs showing the cell coverage area in µm instead of cell numbers (as shown in Fig. 1I+J)(n = 6). Data is presented as mean ±SD (or SEM in live assay graphs)


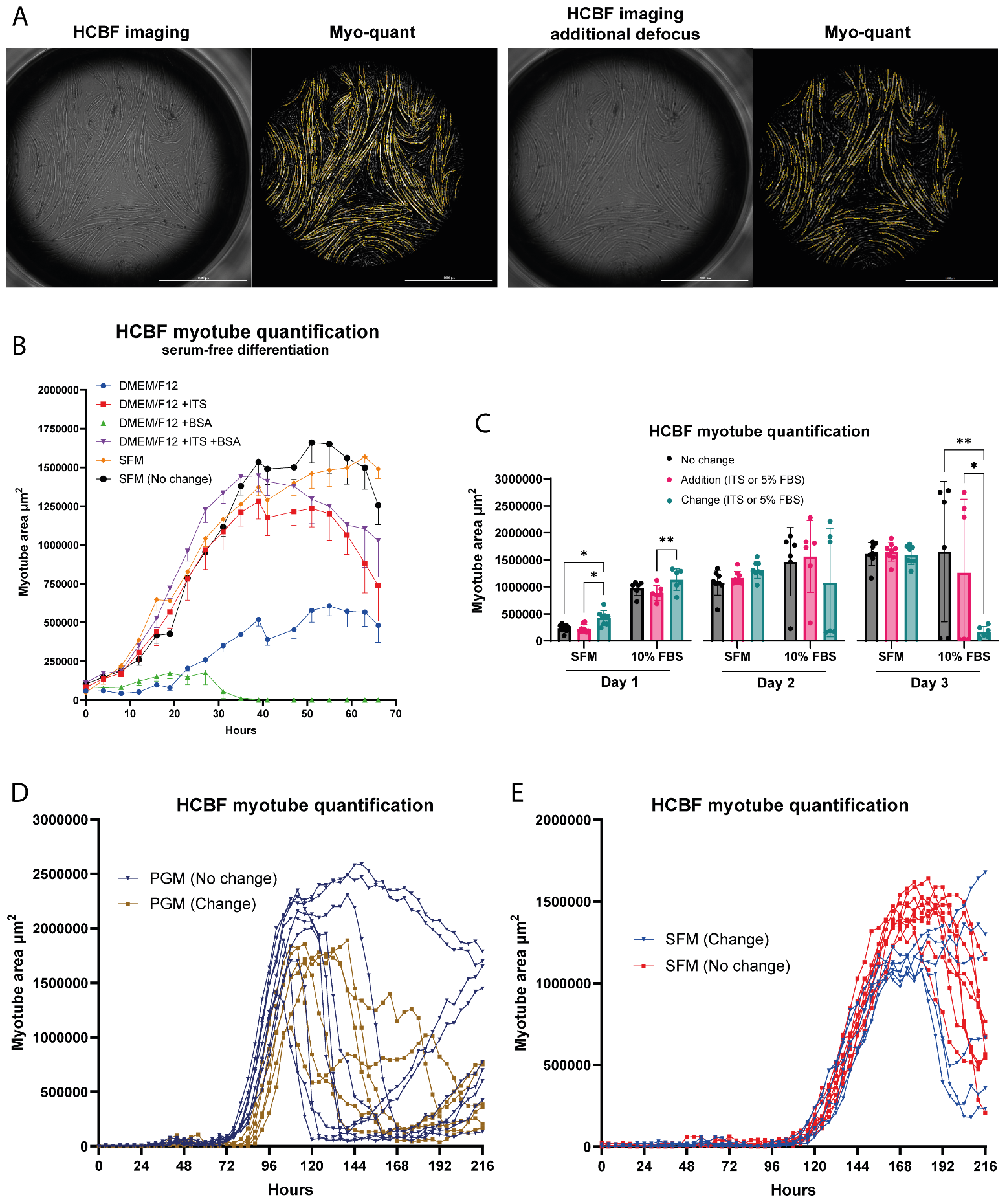


**Supplementary Figure 2: High-contrast brightfield (HCBF) imaging for myotube quantification in 96-well format. A** Representative images of HCBF imaging and myo-quant with two different defocus settings. Left image is the preferred setting used in this study (z-axis = 2070 µm) which includes more myotubes. Right image is a more robust less detailed alternative if samples are difficult and include many single cells into the analyzed myo-quant area (z-axis = 1670 µm). **B** Live assay HCBF myotube quantification (area in µm^2^) of BSCs undergoing myogenic differentiation with different types of serum-free media after 72h of initial proliferation (n = 4). **C** HCBF myotube quantification of BSCs undergoing myogenic differentiation without media change, addition of new media (50 µL/well) or full change of the media at day 1, 2 and 3 after full cell confluency was achieved. For the SFM, media change was DMEM/F12 with ITS only, and for the 10% FBS media change was 5% FBS (n = 6-8). **D** Showing all the individual well replicates (from Fig. 2C) for the PGM samples (with or without media change) (n = 6-9). **E** Showing all the individual well replicates (from Fig. 2C) for the SFM samples (with or without media change) (n = 6-9). Data is presented as mean ±SD (or SEM in live assay graphs). Statistical significance by two-way ANOVA with multiple comparisons in which p < 0.05 (*) and p < 0.01 (**).


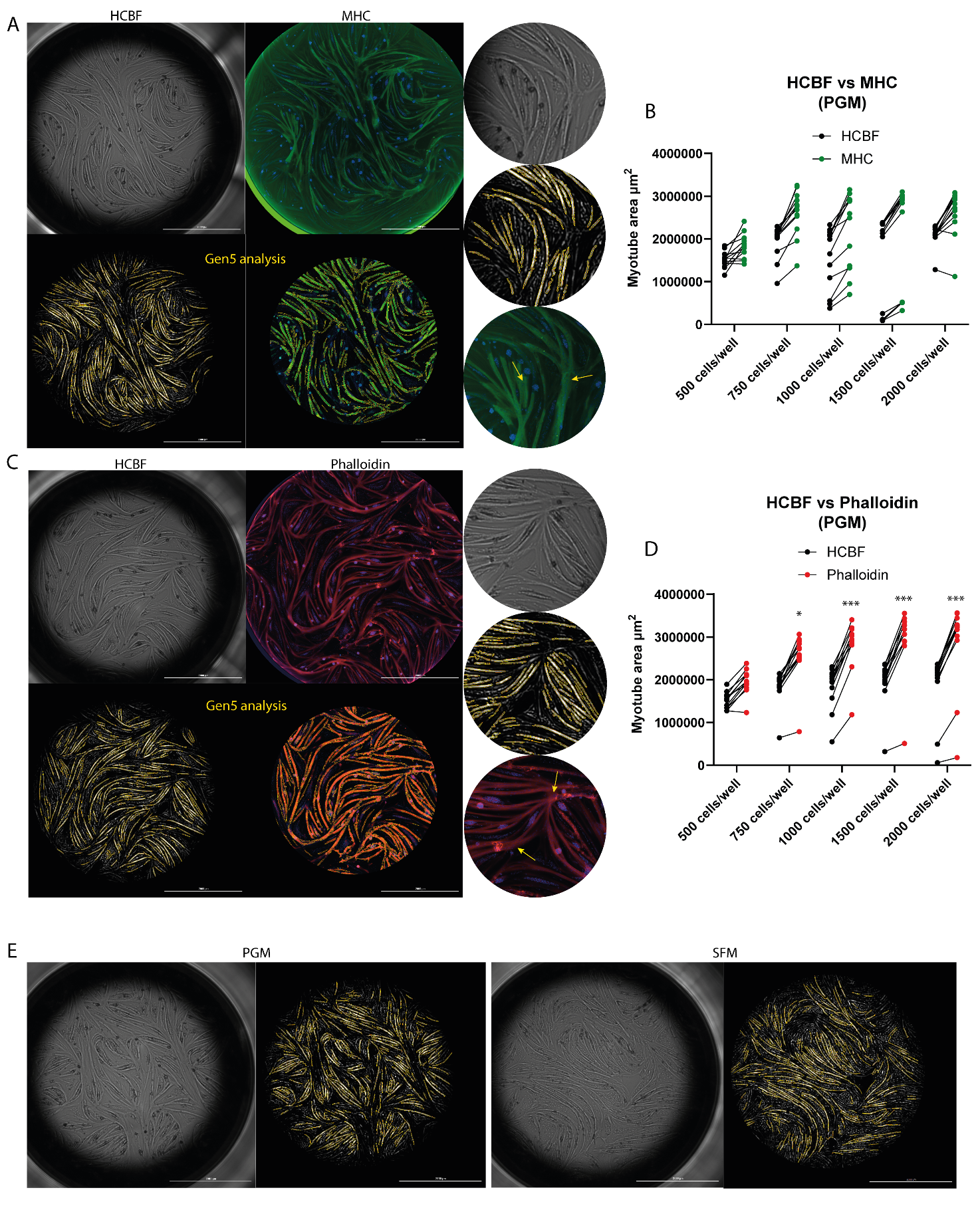


**Supplementary Figure 3: High-contrast brightfield (HCBF) imaging and fluorescence imaging for the quantification of myotubes in PGM media. A** Representative images of HCBF imaging and MHC (myosin-heavy chain) stained fluorescence imaging with and without Gen5 pre-processing and myotube quantification. Arrows indicate spread-out myotube areas that are difficult to quantify with HCBF. **B** Quantification of myotube area with HCBF and MHC stained fluorescence imaging at different starting seeding densities after 4 days of incubation (n = 12). **C** Representative images of HCBF imaging and Phalloidin stained fluorescence imaging with and without Gen5 pre-processing and myotube quantification. Arrows indicate spread-out myotube areas that are difficult to quantify with HCBF. **D** Quantification of myotube area with HCBF and Phalloidin stained fluorescence imaging at different starting seeding densities (n = 12). **E** Representative HCBF images showing the different myotube morphology using either PGM or SFM media. Data is presented as individual replicate well values with interconnected lines between the two compared methods. Statistical significance by two-way ANOVA with multiple comparisons in which p < 0.05 (*) and p < 0.01 (**).


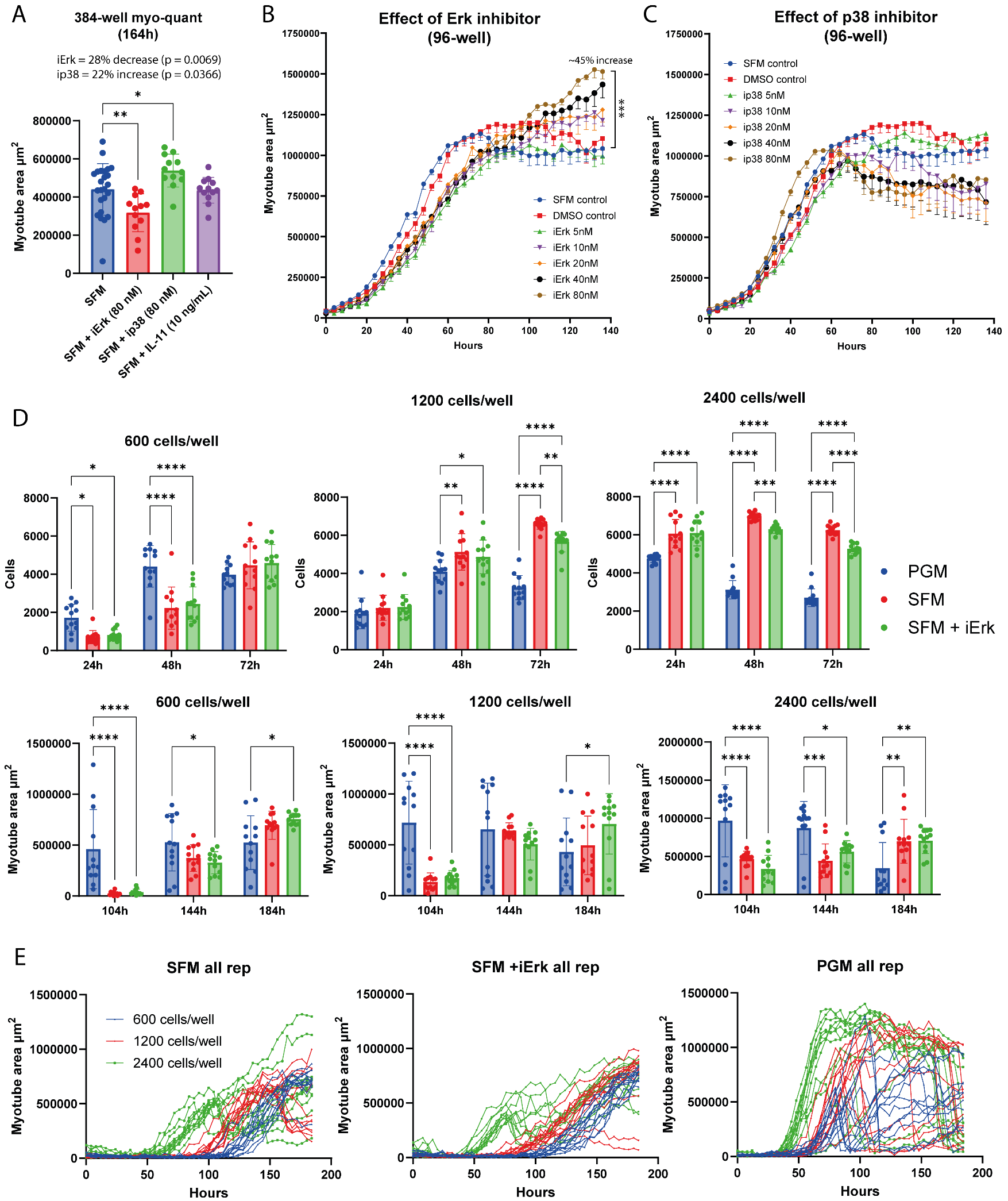


**Supplementary Figure 4: Effect of inhibitors and seeding density on cell growth and differentiation.
A** HCBF myo-quant data from the last time-point in Fig. 3B for the comparison between SFM with or without inhibitors or IL-11 (n = 12-24). **B-C** HCBF myo-quant of differentiating BSCs (after 4 days of initial proliferation) in SFM with or without varying concentrations of Erk or p38 inhibitor without media change in a 96-well format (n = 4-8). **D** HCBF cell count and myo-quant data from three different time-points (related to Fig. 3E-G) for the quantitative comparison between PGM, SFM and SFM + iErk groups with differing seeding densities in the 384-well setup. **E** All replicates are shown for the PGM, SFM and SFM + iErk groups at different seeding densities in a 384-well format (related to Fig. 3E-G) (n = 12). Data is presented as mean ±SD (or SEM in live assay graphs). Statistical significance by one-way (**A**) or two-way (**D**) ANOVA with multiple comparisons in which p < 0.05 (*), p < 0.01 (**), p < 0.001 (***) and p < 0.0001 (****).


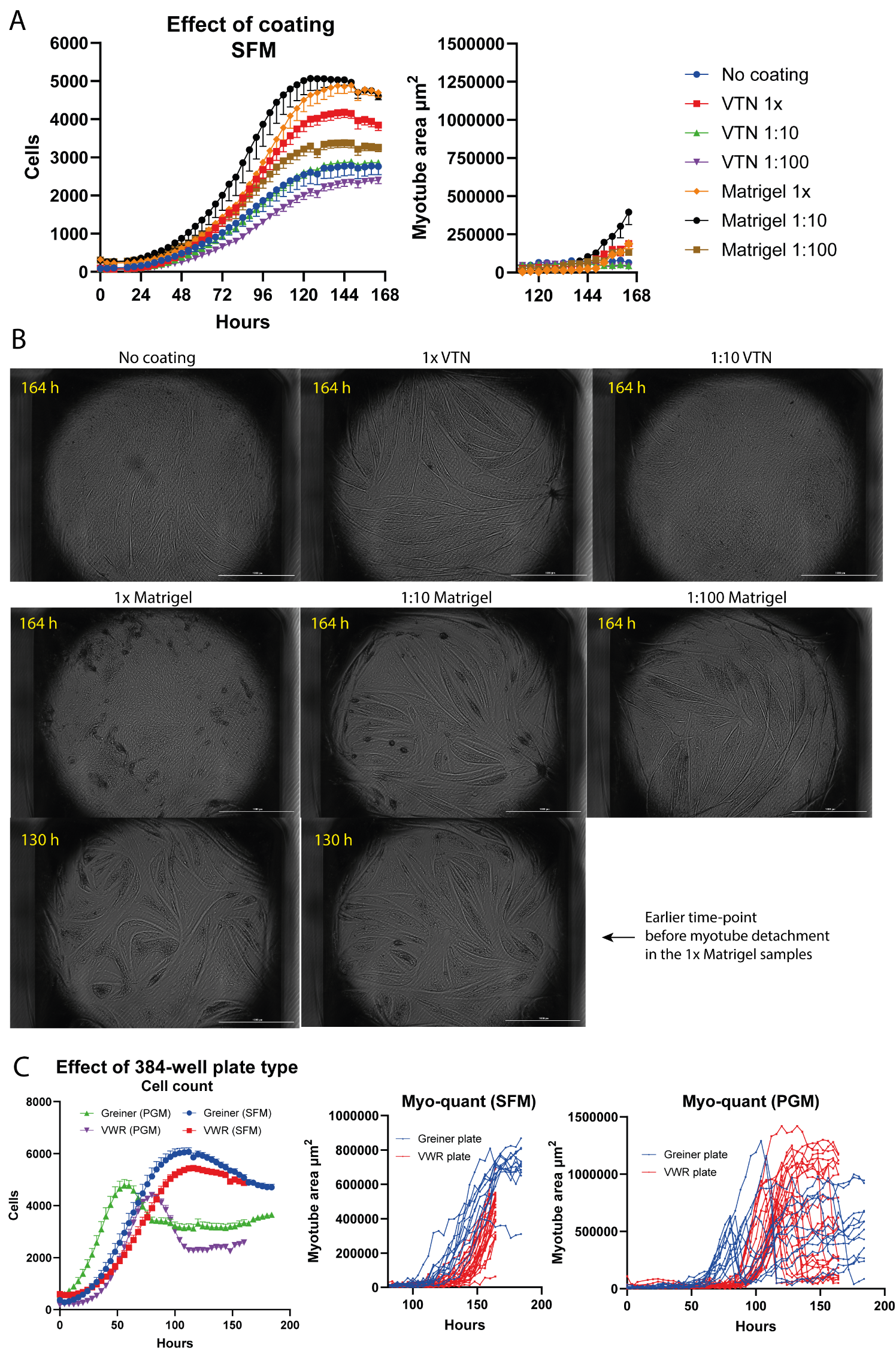


**Supplementary Figure 5: Effect of seeding density and plate manufacturer on the differentiation of BSCs in 384-well plates.
A** HCBF cell count and myo-quant showing the effect of different coatings when grown in SFM in a 384-well format (n = 6). Data is presented as mean ±SEM. **B** Representative HCBF images showing the myotube morphology resulting from the different coatings at the last time-point. The 1x and 1:10 Matrigel samples are shown at the 130-hour time-point as well to highlight the well-formed myotubes in 1x Matrigel that are later detached from the surface. **C** Comparison of the Greiner and VWR 384-well plate types in terms of cell count and myo-quant using SFM or PGM (n = 9-12).

**
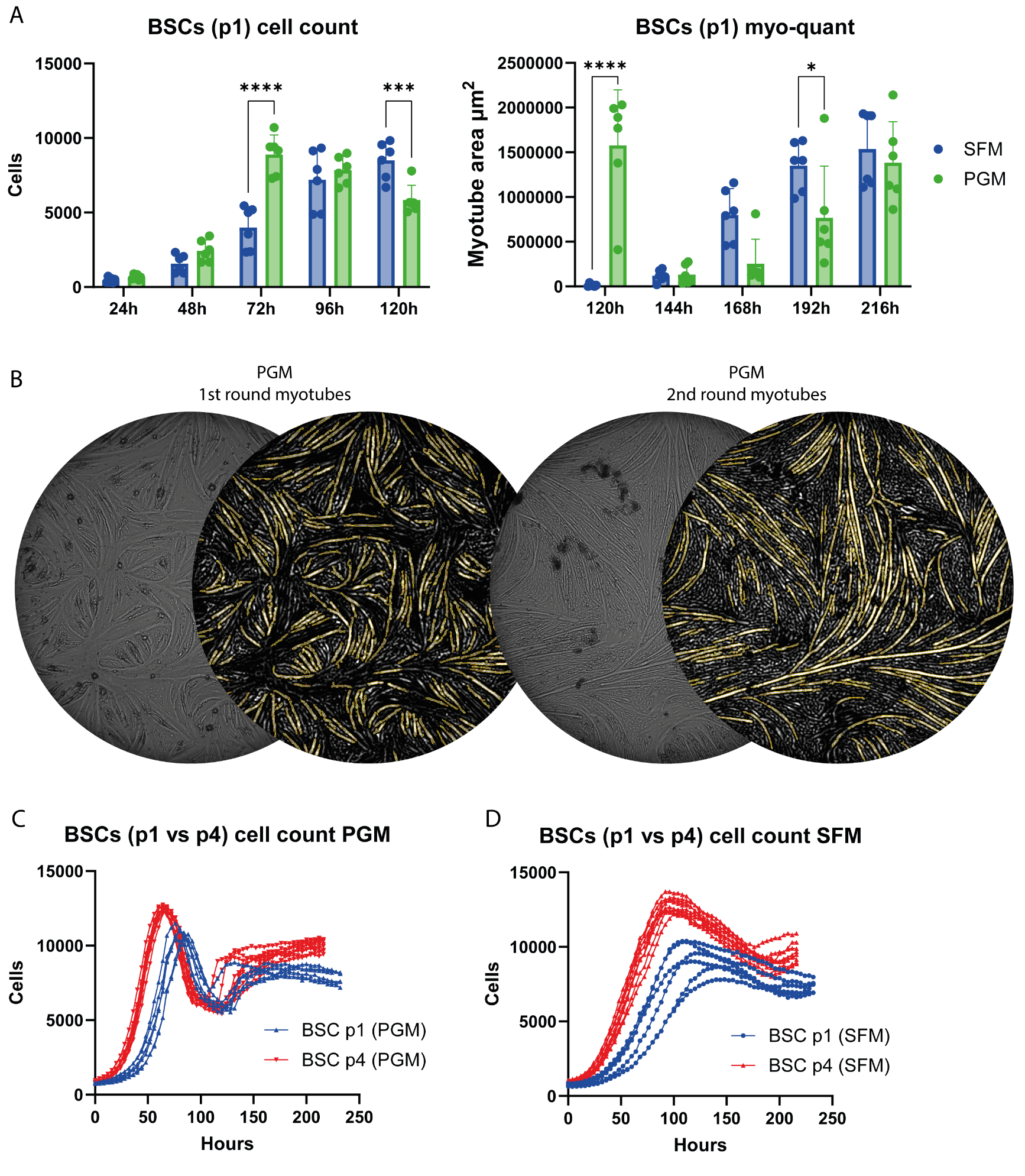
**

**Supplementary Figure 6: Differences between first and second-round myotubes and the passage number on cell growth.
A** Comparison of cell count and myo-quant data between SFM and PGM media at five different time-points (n = 6). **B** Representative HCBF myo-quant images of a BSC (p1) PGM sample with its first and secondary round of myotube formation. **C+D** Comparison between p1 and p4 BSCs in terms of cell count using either PGM or SFM in a 96-well format (n = 6-9). Statistical analysis by two-way ANOVA with multiple comparisons in which p < 0.05 (*), p < 0.001 (***) and p < 0.0001 (****).


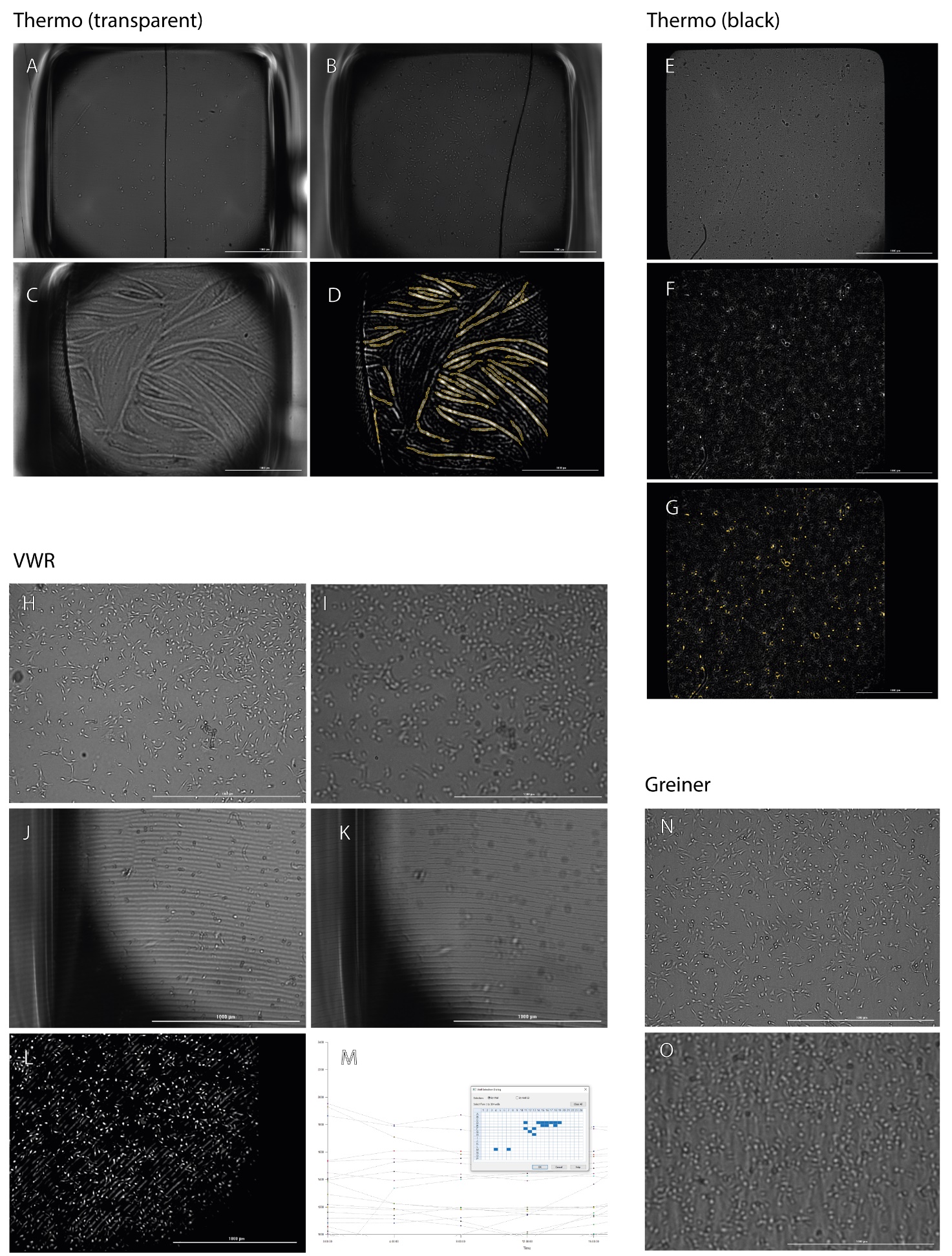


**Supplementary Figure 7: 384-well plate quality issues**
All tested 384-well plates had some type of quality issue/visual artefact within the plastic. Transparent Thermo plates (Cat. No. 164688) (**A-D**) has long lines all the way across the plate going through the well area (probably from the molding process). The placement within the well area was random and was in nearly all wells across the plate. This highly affected myo-quant analysis, yet cell count was minimally affected. Black plates (Cat. No. 142761) (**E-G**) has some granularity throughout the well-bottom plastic making them less clear. This was present in all wells and mostly affected cell counting but also to some extent the myo-quant. The VWR (Cat. No. 10814-226) plate issue (**H-M**) only affected cell counting. This was due to the lines visible in **J** (cell count defocus) and **K** (myo-quant defocus), which only created white lines in the pre-processing of the cell count defocus z-axis setting. The issue was inconsistent across the entire plate and mainly affected the highlighted wells in **M**, which had to be corrected in the data analysis, while most other wells had no issues (**H+I**). Greiner (Cat. No. 781091) plates (**N+O**) had a small degree of granularity/less clear imaging yet only present in the myo-quant defocus setting (**O**) and not the cell count defocus (**N**).
